# A bionic intelligent method combining evolutionary game theory with particle swarm optimization for UAV 3D path planning

**DOI:** 10.3389/fnbot.2026.1857152

**Published:** 2026-06-29

**Authors:** Lixin Jia, Peng Shi

**Affiliations:** School of Astronautics, Beihang University, Beijing, China

**Keywords:** 3D UAV path planning, evolutionary game theory, particle swarm optimization, self-adaptive constraint handling technology, self-adaptive PSO

## Abstract

Recently, UAV path planning in 3D complex environments has attracted increasing attention due to its significance in UAV motion control systems. However, the NP-hard nature of this problem poses significant challenges in generating a high-quality path. To address this issue, this paper proposes an improved self-adaptive particle swarm optimization (ISAPSO) algorithm by integrating the standard PSO 2011 with evolutionary game theory (EGT). Firstly, a novel self-adaptive parameter updating strategy is proposed, which combines the evolutionary stable strategy in EGT with hyperbolic tangent function to balance the exploration and exploitation capabilities of ISAPSO. Subsequently, an ISAPSO-based path planning approach is developed to generate optimal 3D path for UAV in an obstacle-rich environment. To efficiently handle constraints, a novel self-adaptive constraint handling technology is proposed in the developed path planner. Finally, the performance of the proposed ISAPSO is evaluated against six state-of-the-art evolutionary algorithms using 20 test functions. Following the benchmark study, the ISAPSO-based path planner is is validated in different scenarios against six well-known counterparts. The simulation results confirm that the proposed ISPASO outperforms its competitors in the benchmark study at a 90% confidence level. Moreover, the ISPASO-based path planning method dominates its contenders in terms of the path optimality. Therefore, the proposed method could be regarded as a vital alternative in the area of path planning.

## 1 Introduction

Due to its extensive real-world applications, such as rescue, agriculture and military search or attack, unmanned aerial vehicle (UAV) has recently attracted significant attention in robotics community (Xu et al., 2024). As a fundamental and paramount component of the motion planning system, the path planning issue seeks to generate a feasible path for the UAV from a predefined start point to a destination in a complex environment. The quality of the generated path strongly affects the intelligence and automation of the motion planning system of UAV (Lyu and Yang, 2024). Consequently, addressing the UAV path planning issue via different planning methods has become increasingly prevalent in recent years (Yang et al., 2025). Nevertheless, the NP-hard nature of this issue makes it difficult to solve efficently (Tang et al., 2020). To cope with this challenge, numerous researchers have been recently devoted themselves to developing different path planning methods.

To the best knowledge of the authors, the existing path planning methods can be generally classified into two different categories. The first category focuses on traditional planning methods, such as mixed integer linear programming (MILP) (Radmanesh and Kumar, 2016), rapidly-exploring random tree (RRT) (Yang et al., 2014), spares A* (Wang et al., 2014b) and Dijkstra algorithm (Karve and Kapadia, 2020). Despite being mature of the mathematical models and relative easy implementation, the traditional planning algorithms may still struggle with the defect of easily trapping into local optimal when dealing with complicated, nonlinear and non-differential path planning issues (Lyu and Yang, 2024; Zhang et al., 2013).

The aforementioned limitations of classical methods have catalyzed the development of more robust planning approaches based on evolutionary algorithms (EAs). Generally, EAs are bionic intelligent optimization algorithms characterized by their population-based nature and capability for parallel computation (Tang et al., 2017). Owing to these advantages, different EA-based path planning methods, such as ant colony optimization (ACO) algorithm (Hu et al., 2025), genetic algorithm (GA) (Heng and Rahiman, 2025), differential evolution (DE) (Freitas et al., 2024), simulated annealing (SA) (Yu et al., 2024) and black-winged kite algorithm (BKA) (Chen et al., 2025), have been developed and treated as the second but most preferred category in the area of path planning.

As one of the most popular EAs, the particle swarm optimization (PSO) algorithm has been widely employed to address the path planning issue due to its population-based nature. easy of implementation and fast convergence speed (Gupta and Verma, 2023; Gao and Ma, 2024). Capitalizing on these advantages, many different PSO-based path planning algorithms have been recently developed. For instance, an adaptive PSO algorithm by mixing Q-learning with standard PSO has been proposed to handle the UAV path planning problem (Tan et al., 2024). Similarly, a hybrid method combing PSO with GA has been presented to solve the UAV 3D path planning problem (Li et al., 2025). Further examples of PSO applications in UAV path planning can be found in (Liu et al., 2024; Nguyen et al., 2024; Tan et al., 2023). Yet, as a stochastic algorithm, the standard PSO algorithm struggles to dynamically balance exploration and exploitation, which often results in premature convergence of the swarm (Zhang et al., 2025; Raj and Kos, 2023; Tao and Kim, 2024). Therefore, remedying this inherent flaw is essential to further enhancing the performance of PSO.

To efficiently address the UAV 3D path planning problem, this paper focuses on developing a PSO-based path planner that remedies the defects of the standard PSO noted above. To this end, a novel improved self-adaptive PSO algorithm (ISAPSO) is proposed by integrating the standard PSO 2011 (SPSO 2011) (Tang et al., 2020) with evolutionary game theory (EGT) (Leboucher et al., 2016). To achieve a well-balanced exploration-exploitation trade-off, a new self-adaptive parameter updating strategy inspired by the evolutionary stable strategy (ESS) of EGT and the hyperbolic tangent function is developed to tune the three key control parameters of the particle in ISAPSO. As a part of development, this paper completes the establishment of a ISAPSO-based path planning method, in which a modified self-adaptive constraint handling technology based on the feasibility-based rule (Wang et al., 2014a) is presented to easily deal with the constraints of the path planning problem. The performance of the proposed ISAPSO-based path planning method is evaluated under different scenarios by comparing the proposed planner with six well-known heuristic algorithms after a comprehensive benchmark study over 20 benchmark test functions. The non-parameter comparison results over the conducted benchmark study reveal that the ISAPSO algorithm performs significantly better than the six opponents compared at a 90% confidence level. Besides, the developed ISAPSO-based path planning method performs superior to its six peers in terms of the path optimality.

The remainder of this paper is organized as follows. Section 2 presents the problem statement and mathematical model of the path planning issue. Section 3 introduces the proposed ISAPSO algorithm. Section 4 details the ISAPSO-based path planning method, including particle encoding and the modified self-adaptive constraint handling technology. The numerical simulation results and analysis regarding the benchmark study and 3D path planning in different scenarios are presented in Section 5. Section 6 concludes this study and outlines potential directions of future work.

## 2 Description and modeling of UAV path planning

### 2.1 Modeling of the terrain environment

**Figure 1:**
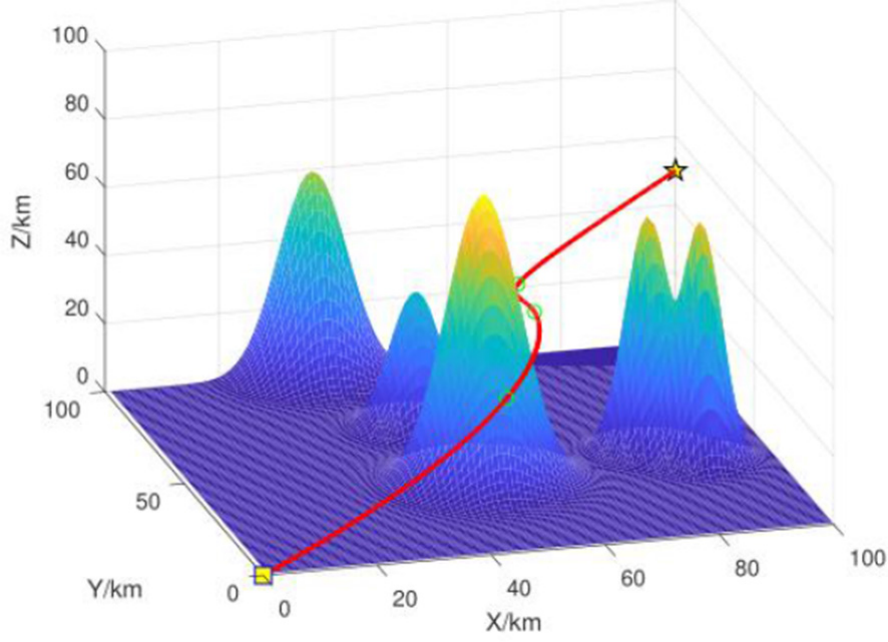
The illustration of the concerned path planning issue.

As displayed in Figure 1, the primary goal of of the path planning problem is to generate a collision-free path connecting the start and destination points for a UAV in a 3D environment. Modeling of the 3D workspace is crucial since it directly impacts the quality of the generated path. In this work, the mountain model is applied to represent the terrain information of the 3D environment due to its simplicity and universality as following (Li et al., 2025):


$$\begin{array}{l} z\left(x,y\right)=\overset{N}{\underset{i=1}{\sum }}z_{i}\exp\left[-{\left(\frac{x-x_{i}}{x_{si}}\right)}^{2}-{\left(\frac{y-y_{i}}{y_{si}}\right)}^{2}\right] \end{array}$$ (1)

where *N* denotes the total number of mountains in the workspace. *x*_*i*_ and *y*_*i*_ stand for the coordinates of the *ith* mountain along the x-axis and y-axis, respectively. *z*_*i*_ represents the peak of the *ith* mountain. *z*(*x, y*) indicates the height of the *ith* mountain. *x*_*si*_ and *y*_*si*_ are slopes of the *ith* mountain along the x-axis and y-axis, respectively.

### 2.2 Mathematical model of path planning

To generate a path similar to that shown in Figure 1, the UAV path is denoted by ordered coordinates in the workspace. To ensure the smoothness of the generated path, this study applies the B-spline curve to model the path due to its simplicity and flexibility in characterizing the path (Zhang et al., 2024; Huang et al., 2024). Let (*S, w*_1_, *w*_2_, *w*_3_, ..., *w*_*n*_, *D*) represent the waypoints along the path, where *S* = (*x*_0_, *y*_0_, *z*_0_), *D* = (*x*_*n*+1_, *y*_*n*+1_, *z*_*n*+1_) and *w*_*i*_ = (*x*_*i*_, *y*_*i*_, *z*_*i*_) stand for the start point, destination point and intermediate waypoint, respectively. Then, the path points along the UAV path can be produced through the B-spline curve as follows:


$$\begin{array}{l} \{\begin{array}{c} x_{i}^{\prime }=\overset{n}{\underset{k=1}{\sum }}B_{k,d}\left(\tau_{i}\right)x_{k}\\ y_{i}^{\prime }=\overset{n}{\underset{k=1}{\sum }}B_{k,d}\left(\tau_{i}\right)y_{k}\\ z_{i}^{\prime }=\overset{n}{\underset{k=1}{\sum }}B_{k,d}\left(\tau_{i}\right)z_{k} \end{array} \end{array}$$ (2)

where *d* is regarded as the measure metric of the smoothness of the curve. Since the smoothness and computation complexity of the curve are increased with the growing of *d*, *d* is empirically set to be 3 in this study by compromising the path smoothness and computation complexity. *B*_*k, d*_(τ_*i*_) represents the blending function and can be determined by the following knot vector:


$$\begin{array}{l} v_{k}=\{\begin{array}{l} 0,\text{if }k\leq d\\ k-d,\text{if }d<k\leq n-1\\ n-d,\text{if }n\leq d \end{array} \end{array}$$ (3)

After determining the knot vector through the above model, the blending function can be mathematically obtained as follows:


$$\begin{array}{l} B_{k,1}\left(\tau_{i}\right)=\{\begin{array}{l} 1,\text{if }v_{k}\leq \tau_{i}<v_{k+1}\\ 0,\text{otherwise} \end{array} \end{array}$$ (4)

$$\begin{array}{l} B_{k,d}\left(\tau_{i}\right)=\frac{\tau_{i}-v_{k}}{v_{k+d-1}-v_{k}}B_{k,d-1}\left(\tau_{i}\right)+\frac{v_{k+d}-\tau_{i}}{v_{k+d}-v_{k+1}}B_{k+1,d-1}\left(\tau_{i}\right) \end{array}$$ (5)

where τ_*i*_ denotes a parameter incrementally increased with a fixed step size within the range of [0, *n* + *d*]. *n* is the total number of points along the path. *d* is the order of the B-spline curve. Recall that *d* = 3 in order to simultaneously consider the path smoothness and computation complexity in this paper.

After representing the smooth path using the cubic B-spline curve depicted above, the path planning problem is formulated as a constrained optimization issue. Since the path length correlates with the UAV's overall fuel consumption in real-world applications, the objective function of the concerned path planning issue is then modeled to minimize the path length. Moreover, the constraints of the path planning issue are determined by the physical features of the UAV and mission execution constraint, which strongly influence the mission execution performance of the UAV. Note that the physical constraints of the UAV mainly include the flight distance and turning angle constraints, respectively. The mission conduction constraint requires the flight safety. Thus, the path planning issue can be mathematically represented as follows:


$$\begin{array}{l} \{\begin{array}{l} \mathrm{Generating}:\;ph=\left[S=w_{0},w_{1},...,w_{n},D=w_{n+1}\right]\\ \mathrm{Minimize}:\;f=\overset{n}{\underset{i=0}{\sum }}∥w_{i+1}-w_{i}∥ \end{array} \end{array}$$ (6)

Subject to:


$$\{\begin{array}{ll} f\leq L^{max} & \left(7\right)\\ \psi_{i}\leq \psi_{i}^{max},\;1\leq i\leq n-1 & \left(8\right)\\ \theta_{i}\leq \theta^{max},\;1\leq i\leq n-1 & \left(9\right)\\ z_{i,interp(x_{i},y_{i})}\leq z_{i}\leq z^{max},\;1\leq i\leq n & \left(10\right) \end{array}$$

where, it has:


$$\begin{array}{l} f=\overset{n}{\underset{i=0}{\sum }}\sqrt{{\left(x_{i+1}-x_{i}\right)}^{2}+{\left(y_{i+1}-y_{i}\right)}^{2}+{\left(z_{i+1}-z_{i}\right)}^{2}} \end{array}$$ (11)

$$\begin{array}{l} \psi_{i}=\arctan\left(\frac{y_{i+2}-y_{i+1}}{x_{i+2}-x_{i+1}}\right)-\arctan\left(\frac{y_{i+1}-y_{i}}{x_{i+1}-x_{i}}\right) \end{array}$$ (12)

$$\begin{array}{l} \theta_{i}=\arctan\left(\frac{z_{i+2}-z_{i+1}}{\sqrt{{\left(x_{i+2}-x_{i+1}\right)}^{2}+{\left(y_{i+2}-y_{i+1}\right)}^{2}}}\right)\\ \;-\arctan\left(\frac{z_{i+1}-z_{i}}{\sqrt{{\left(x_{i+1}-x_{i}\right)}^{2}+{\left(y_{i+1}-y_{i}\right)}^{2}}}\right) \end{array}$$ (13)

where *S* and *D* are the start and destination points of the UAV, respectively. *f* is the objective function denoted by the path length. ||*w*_*i*+1_−*w*_*i*_|| represents the Euclidean distance between waypoint *w*_*i*+1_ and waypoint *w*_*i*_. *n* is the total number of waypoints of the generated path. *L*^*max*^ is the maximum flight path length of the UAV. ψ_*i*_ and ψ^*max*^ are the *ith* turning angle and maximum turning angle of the UAV, respectively. θ_*i*_ and θ^*max*^ are the *ith* climbing slope angle and maximum climbing slope angle of the UAV, respectively. *z*_*i, interp*(_*x*__*i*_, *y*_*i*_)_ stands for the terrain height corresponding to (*x*_*i*_, *y*_*i*_). *z*_*i*_ represents the actual flight height at the *ith* waypoint. *z*^*max*^ is the flight range constraint along z-axis.

## 3 The proposed ISAPSO

Inspired by bird flocking and fish schooling, the standard PSO has been developed since 1995 to handle different global optimization issues. Particularly, PSO is well-suited for solving the UAV 3D path planning problem due to its distinct advantages: (1) it operates directly in continuous space, making it naturally compatible with the continuous coordinate representation of 3D paths; (2) its population-based architecture facilitates parallel computation, thereby significantly reducing computational time; (3) as a stochastic evolutionary algorithm, PSO possesses strong global search powers, making it effective in handling the complexity and non-linearity inherent in the NP-hard path planning problem. However, extensive real-world applications have demonstrated that the performance of the standard PSO has been limited since it struggles to balance exploration and exploitation and thus easily fall into iterative stagnation (Gupta and Verma, 2023; Gao and Ma, 2024). To remedy this deficiency, significant efforts have been made to develop different PSO algorithms. Among those enhanced PSO algorithms, SPSO 2011 would be one of the most preferred version (Tang et al., 2020; Liu and Han, 2017; Zambrano-Bigiarini et al., 2013), which is detailed in the herein contents.

### 3.1 Review of SPSO 2011

**Figure 2:**
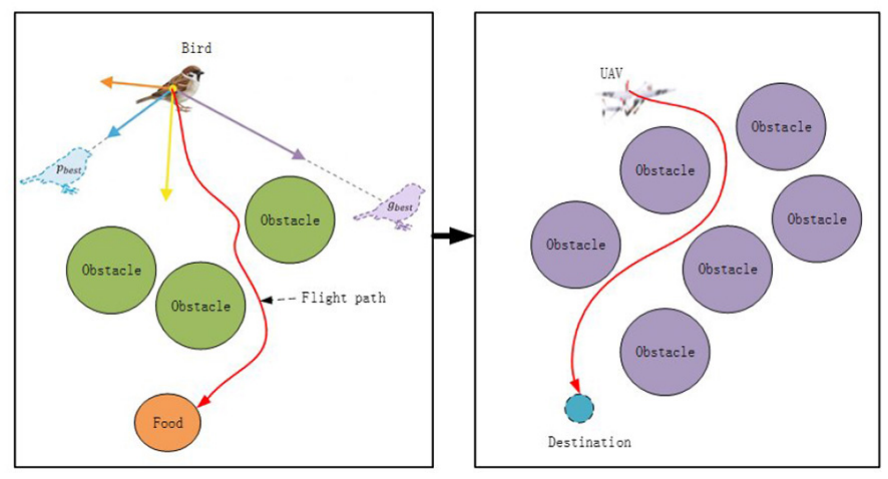
The bionic representation of SPSO 2011 and mapping between UAV path planning issue.

SPSO 2011 is a representative bionic intelligent algorithm, where each agent is regarded as a particle characterized by position and velocity vectors. As displayed in Figure 2, each particle dynamically adjusts its trajectory based on its own flight experience and those of the swarm, converging toward the global optimum while navigating around obstacles within the search space. To overcome the flaw of the standard PSO noted above, SPSO 2011 incorporates random disturbances into the velocity vector. This mechanism ensures that particles maintain non-zero velocities, thereby preserving their exploratory capability and effectively preventing premature convergence (Tang et al., 2020; Liu and Han, 2017; Zambrano-Bigiarini et al., 2013). Due to its noted bionic nature and these advantages, SPSO 2011 is applied in this study to handle the UAV path planning issue.

**Figure 3:**
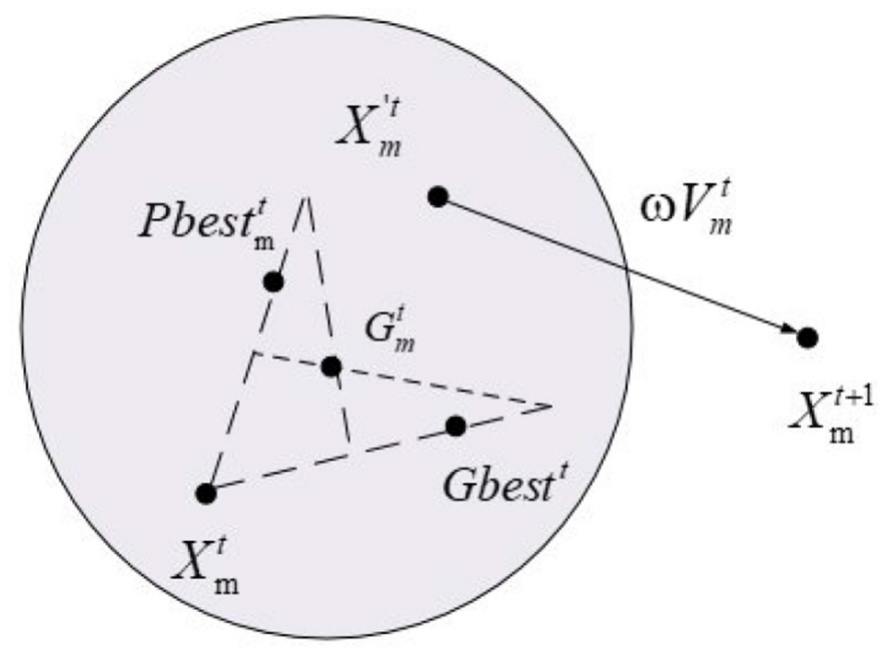
The illustration of SPSO 2011.

Let $X_{m}^{t}$, $Pbest_{m}^{t}$, and *Gbest*^*t*^ denote the current position, the personal best position of the *mth* particle and the global best position of the swarm at the *t*th iteration in SPSO 2011, respectively. As illustrated in Figure 3, an iso-barycenter of gravity denoted as $G_{m}^{t}$ is first defined around the current position $X_{m}^{t}$, the personal best position $Pbest_{m}^{t}$ and the global best position *Gbest*^*t*^ as follows:


$$\begin{array}{l} G_{m}^{t}=X_{m}^{t}+\frac{c_{1}\left(Pbest_{m}^{t}-X_{m}^{t}\right)+c_{2}\left(Gbest^{t}-X_{m}^{t}\right)}{3} \end{array}$$ (14)

where *c*_1_ and *c*_2_ are the cognitive and social acceleration parameters of the particle, respectively.

Then, a random position ${X^{\prime }}_{m}^{t}$ is defined in the hypersphere denoted by *H*(*G*_*m*_(*t*), ||*G*_*m*_(*t*)−*X*_*m*_(*t*)||) and added to the velocity of each particle as follows:


$$\begin{array}{c} V_{m}^{t+1}=\omega V_{m}^{t}+{X^{\prime }}_{m}^{t}-X_{m}^{t} \end{array}$$ (15)

where ω represents the inertia weight parameter of the particle.

Mixing the newly-disturbed velocity defined by Equation 11 with its previous flight experience, each particle updates its position information as follows:


$$\begin{array}{l} X_{m}^{t+1}=V_{m}^{t+1}+X_{m}^{t} \end{array}$$ (16)

where $X_{m}^{t+1}$ is the position of the *mth* particle at iteration *t*+1. In SPSO 2011, the three main control parameters are fixed as constants as follows (Liu and Han, 2017):


$$\begin{array}{l} \{\begin{array}{l} \omega =\frac{1}{2\ln\left(2\right)}\approx 0.7213\\ c_{1}=c_{2}=0.5+\ln\left(2\right)\approx 1.1931 \end{array} \end{array}$$ (17)

It is notable from Equation 11 that since a random position is defined in the hypersphere *H*(*G*_*m*_(*t*), ||*G*_*m*_(*t*)−*X*_*m*_(*t*)||) and added to the velocity of each particle, the particle could be encouraged to keep searching in the solution space with a non-null velocity. This mechanism may prevent SPSO 2011 from prematurely plugging into iterative stagnation, which would promote the optimization performance of this algorithm to some degree. Nevertheless, SPSO 2011 may still suffer from the drawback of unpromising ability in adjusting its exploration and exploitation capabilities since the three main control parameters of the particle are constant, as well as there exists no difference between the cognitive acceleration parameter and the social acceleration parameter (Tang et al., 2020). To overcome the mentioned inherent flaw of SPSO 2011, a newly-developed parameter updating rule is proposed to tune the three key control parameters of the particle in SPSO 2011 by leveraging the EGT. The statements of EGT and novel parameter updating rule are depicted in the herein contents of this paper.

### 3.2 Statement of EGT

EGT is a bionic computational paradigm inspired by biological competition, where the final outcome is determined by the concept of ESS (Leboucher et al., 2016). In EGT, a strategy is treated as an ESS when all individuals in a swarm follow a specific strategy, no other strategies can invade the swarm under the natural selection. Suppose there are a few individuals or players adopting *p*_1_ and some other individuals using strategy *p*_2_. let $f\left(p_{1},\bar{S_{d}}\right)$ represent the payoff, that is, the fitness value of a specific player following strategy *p*_1_. For a minimization optimization problem, *p*_1_ is considered as an ESS if $f\left(p_{2},\bar{S_{d}}\right)>f\left(p_{1},\bar{S_{d}}\right)$ for *p*_2_ ≠ *p*_1_ and any sufficiently small μ (Leboucher et al., 2016). Here, $\bar{S_{d}}$ represents the strategy distribution of the swarm and is computed by:


$$\begin{array}{l} \bar{S_{d}}=\left(1-\mu \right)p_{1}+\mu p_{2} \end{array}$$ (18)

where μ is a sufficiently small parameter, representing the frequency of *p*_2_ in the swarm.

As a common way to describe the strategy interactions among players, the matrix game is stated using the following notations: (1) *e*_*l*_ stands for the pure strategy *l*, where *l* = 1, 2, ..., *M* and *M* denotes the total number of pure strategies; (2) *K* = *f*(*e*_*l*_, *e*_*h*_) is the *M* × *M* payoff matrix; (3) ${▽}^{M}\equiv \left\{p=\left(p_{1},...,p_{M}\right)|\overset{m}{\underset{l=1}{\sum }}p_{l}=1,0\leq p_{l}\leq 1\right\}$ is the set of mixed strategies and represents the probability distribution over the pure strategy *e*_*l*_; (4) *f*(*p, q*) = *pAq*^*T*^ is the payoff, namely, the fitness value of players following the *p*th strategy facing other individuals using strategy *q*. In order to depict the difference between the payoff of strategy *l* and the average payoff of the swarm, the replicator dynamics equation (RDE) in EGT is defined as follows:


$$\begin{array}{l} {ṗ}_{l}=-p_{l}\left(e_{l}Kp^{T}-pKp^{T}\right) \end{array}$$ (19)

where *p*_*l*_ denotes the probability distribution associated with the pure strategy *e*_*l*_. *K* is the payoff matrix.

### 3.3 Proposed Self-adaptive parameter updating rule in ISAPSO

For enhancing the performance of PSO, this paper proposes a novel ISAPSO algorithm based on SPSO 2011 and EGT. The main motivation of the development concerning to the proposed algorithm is to remedy the inherent defect of SPSO 2011 noted above. To achieve this goal, a novel self-adaptive parameter updating strategy inspired by the ESS of EGT and the hyperbolic tangent function is developed to dynamically tune the three aforementioned main control parameters in ISAPSO. To prevent the swarm from early iterative stagnation, particles in the proposed algorithm first stick to the moving rules defined by Equations 10–12 in SPSO 2011 to update their flight experience. Then, a self-adaptive parameter updating strategy is proposed to adjust the three main control parameters of each particle in the proposed algorithm by leveraging the ESS of EGT and the hyperbolic tangent function.

Prior to introducing the proposed parameter updating strategy, the analogy between EGT and ISAPSO is first described as follows: (1) the players in EGT analogize particles in ISAPSO; (2) each particle in ISAPSO adopts three candidate strategies, these are, moving only according to its inertia weight, just following its personal best memory and merely following the global best memory of the swarm, respectively; and (3) the payoff matrix of EGT is consisted by the average performance obtained by each particle in ISAPSO following a specific strategy.

Let *e*_1_, *e*_2_, and *e*_3_ stand for the three aforementioned strategies, respectively. Then, the payoff matrix applied in this study is computed as follows:


$$\begin{array}{l} K=\left\{\begin{array}{ccc} K\left(e_{1}\right) & \frac{K\left(e_{1}\right)-K\left(e_{2}\right)}{2} & \frac{K\left(e_{1}\right)-K\left(e_{3}\right)}{2}\\ \frac{K\left(e_{2}\right)-K\left(e_{1}\right)}{2} & K\left(e_{2}\right) & \frac{K\left(e_{2}\right)-K\left(e_{3}\right)}{2}\\ \frac{K\left(e_{3}\right)-K\left(e_{1}\right)}{2} & \frac{K\left(e_{3}\right)-K\left(e_{2}\right)}{2} & K\left(e_{3}\right) \end{array}\right\} \end{array}$$ (20)

where *K*(*e*_*l*_) (*l* = 1, 2, 3) is the payoff obtained in the case where the particle only adopts the *l*th strategy.

In this study, the ESS indicates the ratio of each strategy when the swarm reaches a stable point and is mathematically defined as follows:


$$\begin{array}{l} E_{ss}\left(t\right)=\left[Z_{1}\left(t\right),Z_{2}\left(t\right),Z_{3}\left(t\right)\right] \end{array}$$ (21)

$$\begin{array}{l} Z_{1}\left(t\right)+Z_{2}\left(t\right)+Z_{3}\left(t\right)=1 \end{array}$$ (22)

At each iteration, the payoff *K*(*e*_*l*_) (*l* = 1, 2, 3) of each particle is gained according to its previous flight experience as follows:


$$\begin{array}{l} K\left(e_{l}\right)=\frac{\overset{t-1}{\underset{t_{1}=1}{\sum }}Z_{l}\left(t_{1}\right)f\left(x\left(t_{1}\right)\right)}{t} \end{array}$$ (23)

where *t* is the iteration number at the current stage. *f*(*x*(*t*_1_)) is the fitness value of particle *x* at the previous iteration *t*_1_.

Once the payoff *K*(*e*_*l*_) (*l* = 1, 2, 3) of each particle is obtained via Equation 19, the payoff matrix is then constructed by Equation 16. Subsequently, the RDE given in Equation 15 is implemented to compute the ESS, that is, *E*_*ss*_(*t*) = [*Z*_1_(*t*), *Z*_2_(*t*), *Z*_3_(*t*)]. By integrating the three ratios *Z*_1_(*t*), *Z*_2_(*t*) and *Z*_3_(*t*) contained in *E*_*ss*_(*t*) with the hyperbolic tangent function, the newly-developed parameter updating strategy in ISAPSO is defined as follows:


$$\begin{array}{l} \omega_{m}^{t+1}=\omega_{s}-\left(\omega_{s}-\omega_{f}\right)\cdot \tanh\left(\frac{\delta_{\omega }t}{\alpha }\right) \end{array}$$ (24)

$$\begin{array}{l} c_{1m}^{t+1}=c_{1s}-\left(c_{1s}-c_{1f}\right)\cdot \tanh\left(\frac{\delta_{c_{1}}t}{\alpha }\right) \end{array}$$ (25)

$$\begin{array}{l} c_{2m}^{t+1}=c_{2s}-\left(c_{2s}-c_{2f}\right)\cdot \tanh\left(-\frac{\delta_{c_{2}}t}{\alpha }\right) \end{array}$$ (26)

where:


$$\begin{array}{l} \delta_{\omega }=\frac{\omega_{s}-\omega_{f}}{t_{max}} \end{array}$$ (27)

$$\begin{array}{l} \delta_{c_{1}}=\frac{c_{1s}-c_{1f}}{t_{max}} \end{array}$$ (28)

$$\begin{array}{l} \delta c_{2}=\frac{c_{2s}-c_{2f}}{t_{max}} \end{array}$$ (29)

$$\begin{array}{l} \alpha =\frac{Z_{1}\left(t\right)+Z_{2}\left(t\right)}{Z_{3}\left(t\right)} \end{array}$$ (30)

$$\begin{array}{l} \tanh\left(x\right)=\frac{e^{x}-e^{-x}}{e^{x}+e^{-x}} \end{array}$$ (31)

where $\omega_{m}^{t+1}$, $c_{1m}^{t+1}$, and $c_{2m}^{t+1}$ are the inertial weight, cognitive and social acceleration parameters of *mth* particle at iteration *t*+1, respectively. *t*_*max*_ is the maximum iteration number. ω_*s*_ and ω_*f*_ stand for the initial and final values of the inertial weight parameter, respectively. *c*_1*s*_ and *c*_1*f*_ are the initial and final values of the cognitive acceleration parameter, respectively. *c*_2*s*_ and *c*_2*f*_ are the initial and final values of the social acceleration parameter, respectively. tanh(*x*) indicates the hyperbolic tangent function.

### 3.4 Analysis of the proposed self-adaptive parameter updating rule

It is apparent from Equations 20–27 that the inertial weight parameter and cognitive acceleration parameter decrease nonlinearly, whereas the the social acceleration parameter of the particle increases nonlinearly with the iteration number increasing. This parameter variation trend indicates that the inertial weight and cognitive acceleration parameter remain relatively large in the early evolution stage, while the social acceleration parameter becomes relatively big in the latter stage of the evolution. Since big values of inertial weight and cognitive acceleration parameter promotes the exploration capability (i.e., the global search power) of the particle and greater social acceleration parameter enhances the exploitation ability (i.e., the local search capability) of the particle (Liu et al., 2024; Nguyen et al., 2024; Tan et al., 2023). Thus, adopting the proposed self-adaptive parameter updating strategy, the global search capability of ISAPSO could be naturally strengthened in the early evolution and its local search capability could be more retained in the late evolution. This natural feature regarding to ISAPSO on one hand could encourage the swarm convergent toward solution space nearby the optimal as soon as possible in the early evolutionary process. On the other hand, the swarm could be encouraged to perform an intensive search in the local solution space containing the optimal solution, thereby enhancing the likelihood of finding the optimal solution.

Apart from the iteration number *t*, the trade-off between the global and local searche abilities of ISAPSO is also modulated by the parameter α. It can be observed from Equations 20–27 that decreasing rates of the inertial weight parameter and cognitive acceleration parameter diminish, while the increasing tendency of the social acceleration parameter also decreases with α increasing. This may imply that the global search capability of ISAPSO would be more maintained with a larger α. Note that a relatively larger α denotes a bigger value of *Z*_1_(*t*) + *Z*_2_(*t*) based on Equation 26. Since a bigger value of *Z*_1_(*t*) + *Z*_2_(*t*) in ESS indicates that the search direction of swarm is more stable when most particles adopt the strategies of following their inertia and personal flight experience, it is reasonable to promote the global search capability of ISAPSO for a relatively bigger α. Contrarily, it is natural to promote the local search ability of ISAPSO for a relatively smaller α since a smaller α indicates a bigger value of *Z*_3_(*t*), which implies the search performance of the proposed algorithm is more stable when the majority of the particles play the strategy based on the global best flight memory of the swarm.

**Figure 4:**
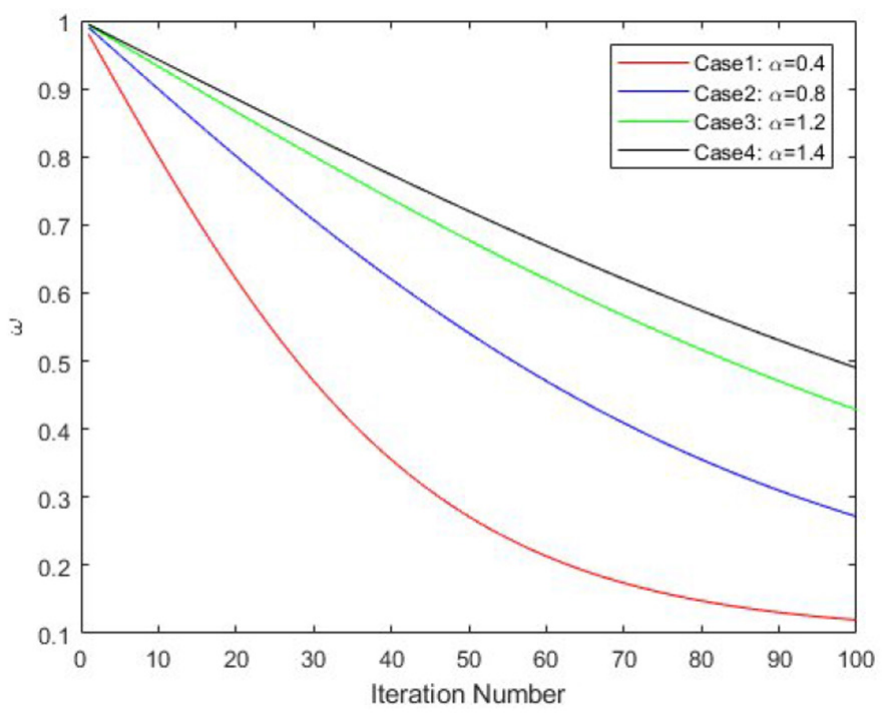
The variation of inertial weight parameter under the self-adaptive updating rule.

**Figure 5:**
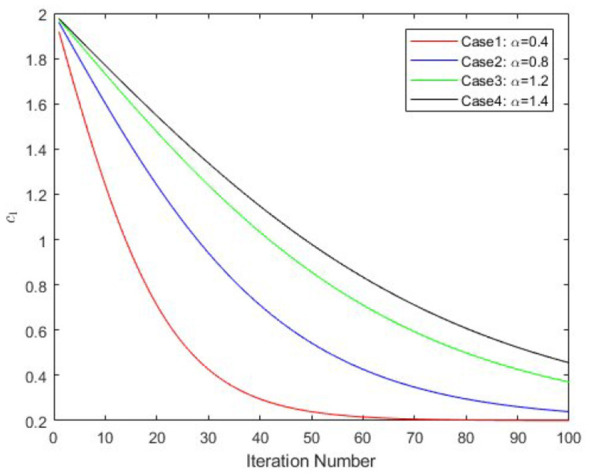
The variation of cognitive acceleration parameter under the self-adaptive updating rule.

**Figure 6:**
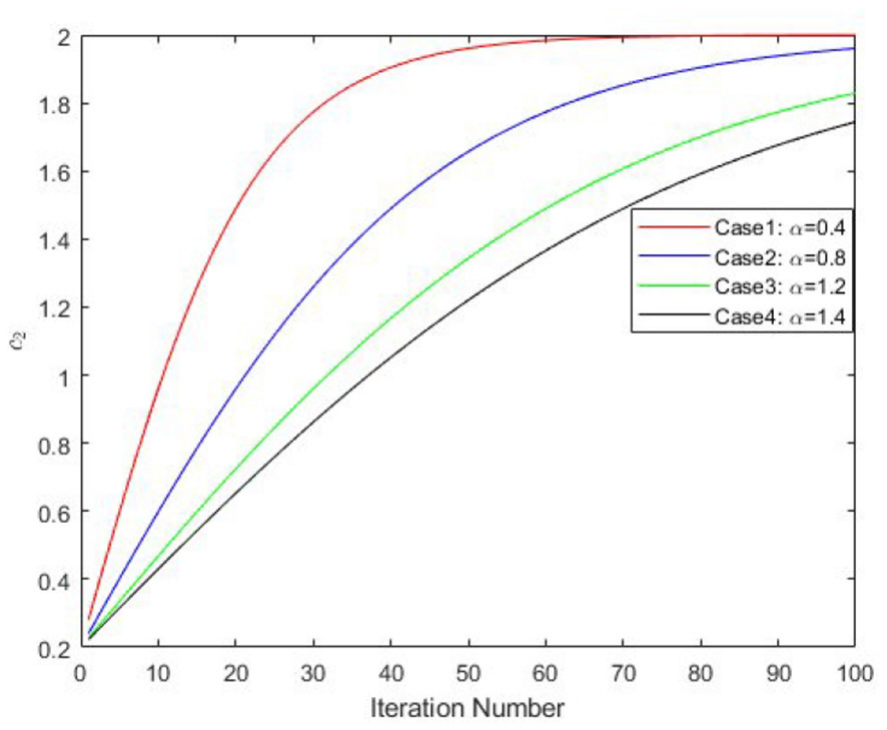
The variation of social acceleration parameter under the self-adaptive updating rule.

Moreover, since the concerned path planning problem is a nonlinear NP-hard issue, adaptively tuning the three key parameters via the developed parameter updating rule may enhance the adaptability of proposed algorithm over the studied path planning problem. Besides, it is well known that the hyperbolic tangent function is a typical saturated activation function with promising convergence speed due to its exponential function characteristic. Employing the hyperbolic tangent manner to update the three key control parameters of the particle may further promote the convergence speed of ISAPSO. Briefly, on one hand, the iterative stagnation issue regarding to ISAPSO would be remedied in the early evolution since this algorithm adopts the same velocity disturbance mechanism introduced in SPSO 2011. On the other hand, the global and local search capabilities of ISAPSO would be well balanced through the developed self-adaptive parameter updating strategy defined by Equations 20–27. All these improvements may promote the optimization performance and convergence speed of the proposed ISPASO over complicated nonlinear optimization issues. The variation tendencies regarding to the three control parameters through the developed self-adaptive updating rule under different values of α are displayed in Figures 4–6 in the case where ω_*s*_ = 1, ω_*f*_ = 0.1, *c*_1*s*_ = *c*_2*f*_ = 2 and *c*_1*f*_ = *c*_2*s*_ = 0.2.

### 3.5 Complexity analysis of ISAPSO

This subsection presents a theoretical analysis of the computational complexity of the proposed ISAPSO algorithm. The computational complexity primarily arises from four sources: (1) initializing the position and velocity of each particle; (2) updating the velocity and position vectors in each iteration; (3) updating the personal best and global best solutions; and (4) adaptively updating the three control parameters via the proposed EGT-based strategy.

Let *NP*, *D*, and *T*_*max*_ denote the swarm size, problem dimensionality and maximum number of iterations, respectively. The initialization phase requires *O*(*NP*·*D*) operations. In each iteration, updating the velocity and position vectors incurs a complexity of *O*(*NP*·*D*). Additionally, the update of personal best and global best positions contributes *O*(*NP*) per iteration. Since the proposed parameter adaptation mechanism employs EGT with *M* candidate strategies, the complexity of updating control parameters across the entire swarm is *O*(*NP*·*M*^2^) per iteration.

Consequently, the total computational complexity (tcc) of the proposed algorithm is expressed as:


$$\begin{array}{l} tcc=O\left(NP\cdot D\right)+O\left(T_{max}\cdot NP\cdot D\right)+O\left(T_{max}\cdot NP\right)+O\left(T_{max}\cdot NP\cdot M^{2}\right) \end{array}$$ (32)

Given that *T*_*max*_ > 1 and *M* is a constant (*M* = 3 in this study), Equation 28 can be simplified by extracting the dominant terms:


$$\begin{array}{l} tcc=O(T_{max}\cdot NP\cdot D)+O(T_{max}\cdot NP\cdot M^{2})=O(T_{max}\cdot NP\cdot (D+M^{2}) \end{array}$$ (33)

It is evident from Equation 29 that the computational complexity of the proposed ISAPSO is primarily governed by the swarm size, the maximum iteration number and the problem dimensionality. Furthermore, the incorporation of EGT for parameter adaptation introduces additionally computational overhead due to the calculation of the payoff matrix and strategy evolution, which is reflected in the *O*(*NP*·*M*^2^) term.

## 4 The ISAPSO-based path planner

This section completes the design of the ISAPSO-based path planning method utilizing the algorithm proposed in Section 3. During this process, particle encoding and constraint handling are two key issues needed to be addressed. These two key technologies are depicted in the following contents of this section.

### 4.1 Encoding particle

Particle encoding aims to establish a mapping between the particle's position vector and a candidate solution to the optimization problem. Since this study focuses on 3D path planning, the position vector of each particle is encoded as a 3 × *n* vector, where *n* denotes the total number of waypoints. The column number of the position vector corresponds to the number of the waypoint. The three position coordinates of each waypoint are orderly encoded based on each column of the corresponding particle's position vector. For guaranteeing each particle to search within the given 3D workspace of UAV, the following saturation method is used to modify each element contained in the position vector of the particle at each iteration as:


$$\begin{array}{l} x_{i}=\{\begin{array}{l} x^{max},\text{ if }x_{i}>x^{max}\\ x^{min},\text{ if }x_{i}<x^{min}\\ x_{i},\;\text{otherwise} \end{array} \end{array}$$ (34)

$$\begin{array}{l} y_{i}=\{\begin{array}{l} y^{max},\text{ if }y_{i}>y^{max}\\ y^{min},\text{ if }y_{i}<y^{min}\\ y_{i},\;\text{otherwise} \end{array} \end{array}$$ (35)

$$\begin{array}{l} z_{i}=\{\begin{array}{l} z^{max},\text{ if }z_{i}>z^{max}\\ z^{min},\text{ if }z_{i}<z^{min}\\ z_{i},\;\text{otherwise} \end{array} \end{array}$$ (36)

where *x*_*i*_, *y*_*i*_, and *z*_*i*_ (*i* = 1, 2, .., *n*) are the three position coordinates of the *i*th waypoint along the genrated path, that is, the three elements in the *i*th column of the position vector of the particle. *x*^*max*^, *y*^*max*^, and *z*^*max*^ are the maximum values of three axes along the 3D workspace system of the UAV, respectively. *x*^*min*^, *y*^*min*^, and *z*^*min*^ are the minimum values of three axes along the 3D workspace system of the UAV, respectively.

### 4.2 Handling constraints

As defined by Equations 7–10, since the path planning problem is subject to physical and flight safety constraints of the UAV, handling these constraints remains the second paramount issue in the ISAPSO-based path planner to guarantee the feasibility of the generated path. To address this issue, a newly-developed constraint handling technology based on the feasibility-based rule is proposed in this paper. In this technology, the violation degree of each particle at each iteration is first computed as follows:


$$\begin{array}{l} CV_{m}^{t}=C_{lm}^{t}+C_{\psi m}^{t}+C_{\theta m}^{t}+C_{sm}^{t} \end{array}$$ (37)

where $CV_{m}^{t}$ is the total violation degree of the *mth* particle at iteration t. $C_{lm}^{t}$,$C_{\psi m}^{t}$, $C_{\theta m}^{t}$, and $C_{sm}^{t}$ are the violation degrees of the flight distance, turning angle, climbing slope angle and the path safety constraints, respectively.

Given the maximum physical constraints of the UAV and the terrain information of the 3D workspace, $C_{lm}^{t}$,$C_{\psi m}^{t}$, $C_{\theta m}^{t}$, and $C_{sm}^{t}$ can be calculated as follows:


$$\begin{array}{l} C_{lm}^{t}=\{\begin{array}{ll} 0 & if\;L_{m}^{t}\leq L^{max}\\ \frac{L_{m}^{t}-L^{max}}{L^{max}} & \mathrm{otherwise} \end{array} \end{array}$$ (38)

where $L_{m}^{t}$ is the path length of particle *m* at iteration t. *L*^*max*^ is a predefined constant, denoting the maximum flight distance of the UAV.


$$\begin{array}{l} C_{\psi m}^{t}=\frac{1}{n}\sum_{i=1}^{n}A_{im}\\ A_{im}=\{\begin{array}{ll} 0 & \text{if }|\psi_{im}^{t}|\leq \psi^{max}\\ 1 & \text{otherwise} \end{array} \end{array}$$ (39)

where *n* is the total number of waypoints along the generated path. $\psi_{im}^{t}$ represents the *ith* turning angle of the path searched by the *mth* particle at iteration *t*, which can be computed via Equation 8. ψ^*max*^ is the maximum turning angle of the UAV.


$$\begin{array}{l} \begin{array}{l} C_{\theta m}^{t}=\frac{1}{n}\overset{n}{\underset{i=1}{\sum }}B_{im}\\ B_{im}=\{\begin{array}{cc} 0 & \mathrm{if}\;|\theta_{im}^{t}|\leq \theta^{max}\\ 1 & \mathrm{otherwise} \end{array} \end{array} \end{array}$$ (40)

where $\theta_{im}^{t}$ indicates the *ith* climbing slope angle of the path found by the *mth* particle at iteration *t*, which can be obtained by Equation 9. θ^*max*^ is the given maximum climbing slope angle of the UAV.


$$\begin{array}{l} C_{sm}^{t}=\frac{1}{n}\sum_{i=1}^{n}C_{im}\\ C_{im}=\{\begin{array}{ll} 0 & \text{if }z_{im}^{t}>z_{i,interp(x_{i},y_{i})}\\ 1 & \text{otherwise} \end{array} \end{array}$$ (41)

where $z_{im}^{t}$ is the height of the *ith* waypoint along the path yielded by the *mth* particle at iteration *t*. *z*_*i, interp*(_*x*__*i*_, *y*_*i*_)_ is the terrain height corresponding to point (*x*_*i*_, *y*_*i*_) and can be gained by the interpolation calculation according to the terrain model defined by Equation 1. (*x*_*i*_, *y*_*i*_) is the position coordinates of the *ith* waypoint of the path searched by the *mth* particle at iteration *t*.

After calculating the total violation degree of the particle using Equations 33–37, an adaptive relaxation variation method is introduced within the proposed constraint-handling scheme of the ISAPSO-based path planner, so as to preserve population diversity. To implement this strategy, a relaxation variable is first defined and computed as follows:


$$\begin{array}{l} CV_{r}^{t}=CV_{0}\cdot \left(1-\frac{N_{f}^{t}}{NP}\right)\cdot \exp\left(-t\right) \end{array}$$ (42)

where $CV_{r}^{t}$ is the value of the relaxation variable at iteration *t*. $N_{f}^{t}$ is the total number of feasible particles at iteration *t*. *NP* is the total number of particles in the ISAPSO-based planner. *CV*_0_ is the average violation constraint degree regarding to the particle swarm at the initial iteration. After initializing the swarm and evaluating each particle's total constraint violation degree via Equations 33–37, *CV*_0_ can be computed as follows:


$$\begin{array}{l} CV_{0}=\frac{1}{NP}\overset{NP}{\underset{m=1}{\sum }}CV_{m}^{0} \end{array}$$ (43)

**Figure 7:**
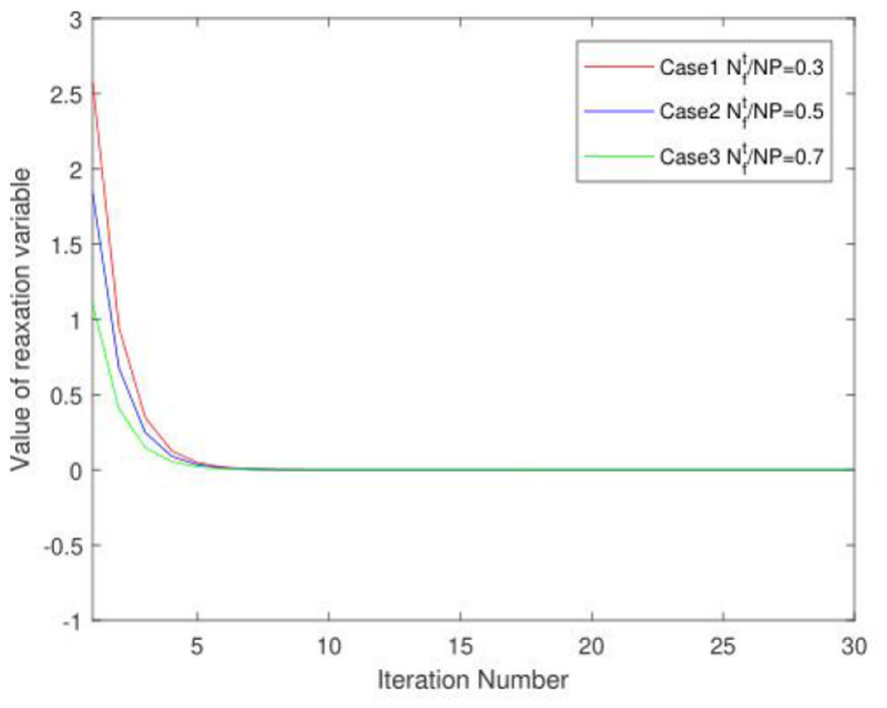
The variation curves of the relaxation variable under different $N_{f}^{t}/NP$ cases.

Figure 7 displays the variation curves of the relaxation variable in the case where $\frac{N_{f}^{t}}{NP}=0.3,0.5,0.7$ and *CV*_0_ = 8, respectively. After iteratively computing the relaxation variable following Equations 38, 39, each particle at each iteration is considered and defined as a feasible particle (or solution) in the case where $CV_{m}^{t}\leq CV_{r}^{t}$. Followafter redefining the feasibility of the particle at each iteration, the following feasibility-based rule is applied in the developed constraint handling technology to select the elite solution between any two candidate solutions in the ISAPSO-based planner as: (1) for any two feasible solutions, the solution owing the smaller fitness value dominates the one having greater fitness value; (2) for any feasible solution and any infeasible solution, the feasible solution dominates the infeasible solution; (3) for any two infeasible solutions, the solution with smaller violation degree dominates the solution with greater violation degree.

It is worth noting that the developed self-adaptive constraint handling technology evaluates the fitness values and constraint violation degrees of any two candidate solutions independently, without introducing additional control parameters. Thus, the computational burden of the ISAPSO-based planner in managing constraints could be effectively reduced. Besides, since some real infeasible solutions may still contain valuable information about the solution space, permitting them to advance to the next iteration may help to diversify the swarm, thereby increasing the likelihood of locating the global optimum.

To achieve the aforementioned objective, a self-adaptive variation strategy defined by Equations 38, 39 is introduced within the proposed constraint handling technology to dynamically redefine the feasibility of each particle. Adopting this strategy, some real infeasible solutions could be reclassified as feasible and permitted into next generations. This would naturally contribute to maintaining population diversity and enhance the capability of generating high-quality paths. Moreover, as evidenced by Equations 38, 39 and Figure 7, the relaxation variable decreases nonlinearly with the iteration number increasing and converges to zero in the latter stage of the evolution. This may indicate that the feasibility criteria become progressively stricter. Therefore, infeasible solutions are more likely to be retained during the early evolutionary phase to promote diversity, whereas most are discarded in the later stages to ensure that the final path strictly satisfies the UAV's physical and safety constraints. To summarize, the proposed self-adaptive constraint handling method may not only address constraints without imposing additional computation burden on the optimizer, but also diversify the swarm, therefore enhancing the probability of obtaining high-quality path for the UAV.

### 4.3 The framework of the ISAPSO-based path planner

This subsection outlines the overall framework of the ISAPSO-based path planning method for solving the path planning problem defined by Equations 6–10. Let *NP* and *t*_*max*_ denote the swarm size and maximum iteration number of ISAPSO, respectively. The algorithmic steps of ISAPSO-based path planner are summarized and shown in Table 1. For a minimization problem, the ways of updating the personal best and global best solutions at the following algorithmic steps can be mathematically given as follows:


$$\begin{array}{l} Pbest_{m}^{t}=\{\begin{array}{l} x_{m}^{t},\text{if }f\left(x_{m}^{t}\right)≼f\left(Pbest_{m}^{\left(t-1\right)}\right)\\ Pbest_{m}^{\left(t-1\right)},\;\text{otherwise} \end{array} \end{array}$$ (44)

$$\begin{array}{l} Gbest^{t}=\{\begin{array}{l} Pbest_{m}^{t},\text{if }f\left(Pbest_{m}^{t}\right)≼f\left(Gbest^{\left(t-1\right)}\right)\\ Gbest^{\left(t-1\right)},\;\text{otherwise} \end{array} \end{array}$$ (45)

where $x_{m}^{t}$ is the position vector of particle *m* at iteration *t*. $Pbest_{m}^{t}$ denotes the personal best solution of particle *m* at iteration *t*. *Gbest*^*t*^ is the global best solution of the swarm at iteration *t*. *f*(·) is the fitness value corresponding to different position vectors. $f\left(x_{m}^{t}\right)≼f\left(Pbest_{m}^{\left(t-1\right)}\right)$ indicates that solution $x_{m}^{t}$ dominates solution $Pbest_{m}^{\left(t-1\right)}$ according to the developed constraint handling technology.

**Table 1:** The algorithmic steps of the ISAPSO-based path planning method.

1. Initialize the particle swarm and set the needed simulation parameters
2. Calculate the initial fitness value via Equation 6 and violation degree via Equations 33–37 of each particle
3. Obtain the average violation degree of the swarm at the initial iteration based on Equation 39
4. Randomly initialize the payoffs of *K*(*e*1), *K*(*e*2) and *K*(*e*3) via Equation 18
5. Gain the initial *Pbest* of each particle and *Gbest* via the developed self-adaptive constraint handling method
6. **while** *t* ≤ *t*_*max*_ **do**
7. **for** *m* ≤ *NP* **do**
8. Update the velocity and position vector of each particle *m* using Equations 10–12
9. Modify each element of the *mth* particle's position vector using Equations 30–32
10. Calculate the fitness value via Equation 6 and violation degree of particle *m* via Equations 33–Equation 37
11. Redefine the feasibility of the *mth* particle by comparing its violation degree with the relaxation variable
12. Obtain the total number of feasible particles at iteration *t*
13. Update *Pbest* for the particle and *Gbest* of the swarm using the developed self-adaptive constraint handling method
14. Update the relaxation variable according to Equation 38
15. Calculate the payoff *K*(*el*) of the particle by Equation 19
16. Obtain the payoff matrix based on Equation 16
17. Calculate the *Z*_1_(*t*), *Z*_2_(*t*) and *Z*_3_(*t*) based on Equation 15
18. Update the three control parameters of particle *m* via Equations 20–27
19. **end for**
20. **end while**
21. Output *Gbest* as the final generated 3D path

## 5 Numerical simulation and result analysis

To verify the proposed method, three distinct sets of numerical simulations are conducted in this paper. The first numerical simulation employs ablation experiments to validate the efficacy of the velocity perturbation mechanism in SPSO 2011 and the self-adaptive parameter tuning strategy in the proposed ISAPSO. The second set focuses on evaluating the performance of the ISAPSO algorithm across 20 benchmark test functions. Following the benchmark tests, the ISAPSO-based path planning method is further assessed under two different 3D path planning scenarios. All the conducted simulation studies are described in detail in the following sections.

### 5.1 Ablation experiment

The purpose of this ablation study is to simply verify the effectiveness of the random velocity perturbation mechanism in SPSO 2011 and the adaptive parameter adjustment mechanism in the proposed ISAPSO. It is worth noting that the primary distinction between SPSO 2011 and the standard PSO lies in the introduction of the random velocity perturbation mechanism. Furthermore, the key difference between ISAPSO and SPSO 2011 is that ISAPSO incorporates an adaptive parameter tuning law into the SPSO 2011 framework. Therefore, in the conducted ablation experiments, the performance of the standard PSO, SPSO 2011 and ISAPSO is comparatively evaluated through numerical simulations on two benchmark functions. The characteristics of these benchmark functions are summarized in Table 2.

**Table 2:** Information of the benchmarks used in the ablation study (“D” stands for the dimension).

Name	Search space	D	Optimal	Model
Shifted Schwefel	[−10, 10]	30	0	$\begin{array}{l} f\left(\text{x}\right)=\\ \overset{n}{\underset{i=1}{\sum }}|x_{i}|+\prod_{i=1}^{n}|x_{i}| \end{array}$
Rastrigin	[−5.12, 5.12]	30	0	$\begin{array}{l} f\left(\text{x}\right)=10n+\overset{n}{\underset{i=1}{\sum }}\\ \left[x_{i}^{2}-10\cos\left(2\pi x_{i}\right)\right] \end{array}$

In the conducted ablation experiments, each algorithm is independently run 10 times on each benchmark function. For each run, the population size and the maximum iteration number of each algorithm are set to 50 and 500, respectively. After completing the runs for each benchmark function, the average solution accuracy, denoted by *E*_*mean*_, is adopted as the metric to evaluate the performance of the considered algorithms. To ensure the convergence rate of the proposed method, the initial and final values of the control parameters in the proposed self-adaptive parameter updating strategy are empirically set as ω_*s*_ = 0.9, ω_*f*_ = 0.3, *c*_1*s*_ = *c*_2*f*_ = 2 and *c*_1*f*_ = *c*_2*s*_ = 0.2.

**Figure 8:**
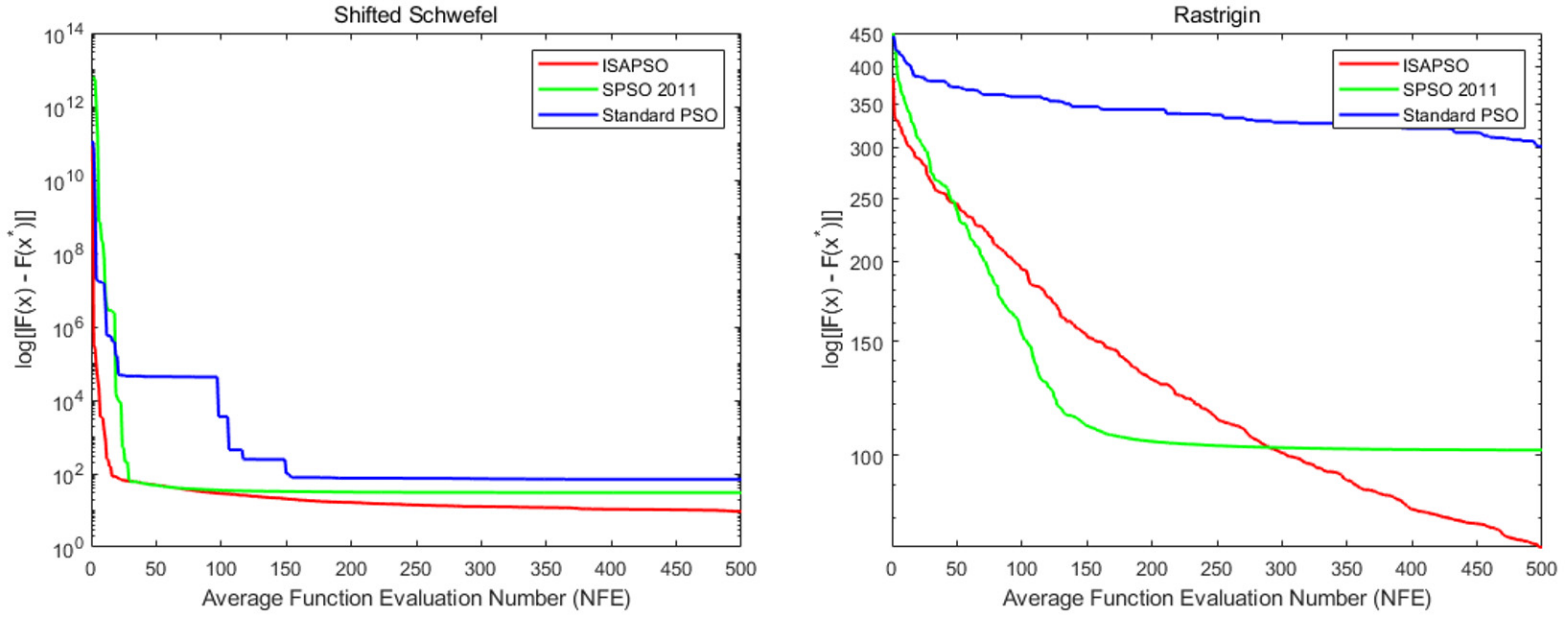
The average fitness value of each algorithm on each benchmark function in the ablation study.

The statistical results of *E*_mean_ obtained by each algorithm on each benchmark function in the conducted ablation study are given in Table 3. The average fitness value of each algorithm on each benchmark function is shown in Figure 8. As shown in Table Table 3, the results of the ablation experiments shown in Table 3 indicate that the performance ranking of the three algorithms is ISAPSO, SPSO 2011 and Standard PSO in terms of average solution quality. It is worth noting that the primary distinction between SPSO 2011 and Standard PSO is the introduction of a velocity perturbation mechanism designed to prevent premature convergence of the swarm. Furthermore, the key difference between ISAPSO and SPSO 2011 lies in the incorporation of the proposed self-adaptive parameter updating strategy. Therefore, the data presented in Table 3 can reflect the efficacy of the velocity perturbation mechanism in SPSO 2011 and the parameter updating strategy proposed in ISAPSO to some extent.

**Table 3:** Statistical results of *E*_mean_ obtained by each algorithm on each benchmark function in the ablation experiment.

Fun.	Items	Algorithms		
		Standard PSO	SPSO 2011	ISAPSO
Shifted Schwefel	*E*_best_	5.6147E+01	1.9645E+01	8.4131E-01
	*E*_mean_	7.0606E+01	3.0351E+01	**9.2905E+00**
	*E*_worst_	1.0679E+01	4.0353E+01	2.0904E+01
	*Std*.	1.9242E+01	7.4662E+00	7.0610E+00
Rastrigin	*E*_best_	2.5945E+02	5.5886E+01	4.4190E+01
	*E*_mean_	3.0099E+02	1.0193E+01	**7.2125E+01**
	*E*_worst_	3.4785E+02	1.5785E+02	1.2617E+02
	*Std*.	2.9072E+01	3.2190E+01	2.5362E+01

The bold values provided in these tables denote the best value obtained by each method of the corresponding item.

### 5.2 Benchmark study

Following the ablation study to simply verify the effectiveness of the proposed parameter updating strategy in ISAPSO, the performance of this algorithm is further evaluated using 20 benchmark test functions, as listed in Table 4. For a rigorous comparison, ISAPSO is benchmarked against six well-known evolutionary algorithms: IPSO (Raj and Kos, 2023), SPSO 2011 (Liu and Han, 2017), EGPSO (Leboucher et al., 2016), GWO (Yang et al., 2025), FDE (Gupta et al., 2025) and quantum technology-based genetic algorithm (QGA) (Wu, 2024). For comprehensive comparison, a Monte-carlo experiment test with 25 runs is conducted for each algorithm on every benchmark function. In each run, the swarm size and maximum iteration number of each method are empirically set to be 50 and 1*E*+05, respectively. The evolutionary process terminates when either the maximum iteration number is reached or the solution error meets the predefined accuracy threshold specified in Table 4. For each benchmark test function, the solution accuracy metric denoted by *E*_*mean*_ is applied as a metric to verify the performance of each considered algorithm. Furthermore, the initial and final values of the control parameters in ISAPSO are set consistent with those shown in SubSection 5.1.

**Table 4:** Information of selected benchmark test functions (“D” stands for the dimension and “ξ” indicates the accuracy level).

Function item and group	Function name	Search space	Optimal	D	ξ
Group1: separable					
*F*_1_	Rosebrock	[−30, 30]	0	30	1E-06
*F*_2_	Rastrigin	[−5.12, 5.12]	0	30	1E-06
*F*_3_	Ackely	[−32, 32]	0	30	1E-06
*F*_4_	Griewwank	[−600, 600]	0	30	1E-06
Group2: non-separable					
*F*_5_	Six-hump Camel-back	[−5, 5]	−1.032	2	1E-06
*F*_6_	Golden-Price	[−2, 2]	3	2	1E-02
*F*_7_	Schaffer *F*_6_	[−100, 100]	0	2	1E-02
Group3: CEC 2005					
*F*_8_	Shifited sphere	[−100, 100]	−450	30	1E-02
*F*_9_	Shifted Schwefel 1.2	[−100, 100]	−450	30	1E-02
*F*_10_	Shifted rotated high conditioned elliptic	[−100, 100]	−450	30	1E-02
*F*_11_	Shifted Schwefel 1.2 with noise in fitness	[−100, 100]	−450	30	1E-02
*F*_12_	Schwefel's problem 2.6	[−100,100]	−310	30	1E-02
*F*_13_	Shifted Rosenbrock	[−100, 100]	390	30	1E-02
*F*_14_	Shifted rotated Griewank without bounds	[0, 600]	−180	30	1E-02
*F*_15_	Shifted rotated Ackley with global optimum on bounds	[−32, 32]	−140	30	1E-02
*F*_16_	Shifted Rastrigin	[−5, 5]	−330	30	1E-02
*F*_17_	Shifted rotated Rastrigin	[−5, 5]	−330	30	1E-01
*F*_18_	Shifted rotated Weierstrass	[−0.5, 0.5]	90	30	1E-01
*F*_19_	Schwefel's problem 2.13	[−π,π]	−460	30	1E-10
*F*_20_	Rosenbrock's function *F*_8_*F*_2_	[−5, 5]	−130	30	1E-10

Following the Monte Carlo simulations described above, the stochastic results regarding solution accuracy obtained by different algorithms for each benchmark function are summarized in Tables 5, 6, where the best average result of *E*_*mean*_ yielded by each method for each test function is highlighted in boldface. The average fitness curve of each method for each test function is illustrated in Appendix.

**Table 5:** The statistical results regarding to *E*_*mean*_ gained by each algorithm for each benchmark.

Fun.	Items	Methods						
		SPSO 2011	QGA	EGPSO	FDE	GWO	IPSO	Proposed
	*E*_*best*_	3.78E-07	4.95E-07	4.17E-07	5.29E-07	4.22E-07	2.91E-07	2.90E-07
*F*_1_	*E*_*mean*_	8.92E-07	8.12E-07	7.42E-07	8.52E-07	8.09E-07	6.86E-07	**6.21E-07**
	*E*_*worst*_	1.25E-06	9.99E-07	9.98E-07	9.94E-07	9.91E-07	9.67E-07	7.99E-07
	*Std*.	2.45E-07	1.45E-07	1.74E-07	1.50E-07	1.49E-07	1.88E-07	1.31E-07
*F*_2_	*E*_*best*_	7.92E-02	7.28E-07	4.49E-07	4.15E-07	2.16E-07	3.94E-07	3.93E-07
	*E*_*mean*_	1.01E-01	9.34E-07	9.05E-07	7.97E-07	7.94E-06	7.57E-07	**6.27E-07**
	*E*_*worst*_	8.54E-01	9.98E-07	9.98E-07	9.99E-07	1.11E-06	9.49E-07	7.61E-07
	*Std*.	2.14E-01	6.33E-07	1.24E-07	1.84E-07	2.44E-05	1.74E-07	1.04E-07
*F*_3_	*E*_*best*_	1.94E+01	5.88E+02	6.75E+01	5.48E+02	9.86E+00	1.28E+01	1.54E-02
	*E*_*mean*_	1.04E+02	8.15E+02	1.03E+02	2.91E+02	2.71E+01	3.52E+01	**2.56E+00**
	*E*_*worst*_	2.63E+02	3.32E+03	3.21E+02	1.26E+03	5.85E+02	7.61E+01	3.46E+01
	*Std*.	6.85E+01	8.69E+02	6.82E+01	2.42E+02	1.16E+02	1.51E+01	7.26E+00
*F*_4_	*E*_*best*_	5.42E+01	3.20E-06	2.64E-07	7.46E-07	5.54E-07	1.762E-07	6.49E-07
	*E*_*mean*_	5.24E+00	4.85E-04	6.77E-05	9.08E-07	**8.65E-07**	4.51E-05	9.07E-07
	*E*_*worst*_	1.44E+01	2.97E-03	1.62E-03	9.99E-07	9.94E-07	1.08E-03	9.97E-07
	*Std*.	3.50E+01	7.86E-04	3.24E-04	9.05E-07	1.084E-07	2.16E-04	7.43E-07
*F*_5_	*E*_*best*_	3.11E+01	8.62E+00	2.05E-02	1.14E-02	3.59E-03	8.45E-07	8.17E-07
	*E*_*mean*_	1.90E+03	2.12E+02	4.31E-01	2.41E-01	7.12E-03	3.03E-04	**9.39E-07**
	*E*_*worst*_	1.05E+04	7.13E+02	2.65E+00	1.48E+00	1.02E-02	7.55E-03	9.95E-07
	*Std*.	2.25E+03	1.91E+02E	6.06E-01	3.39E-01	1.90E-03	1.51E-03	4.28E-08
*F*_6_	*E*_*best*_	8.67E+01	4.59E-01	2.86E-01	9.67E-01	6.35E-02	2.58E-03	2.56E-03
	*E*_*mean*_	9.07E+02	9.92E+01	1.68E+01	3.29E+00	2.27E-02	1.04E-02	**9.16E-03**
	*E*_*worst*_	4.17E+02	4.01E+02	1.08E+02	3.81E+01	9.68E-02	7.34E-02	3.90E-02
	*Std*.	1.22E+02	1.29E+01	3.18E+01	8.04E+00	1.63E-02	1.34E-02	6.60E-02
*F*_7_	*E*_*best*_	156E+03	9.05E+02	1.27E+03	1.21E+02	5.07E-03	7.58E+01	2.53E-03
	*E*_*mean*_	1.58E+03	9.09E+02	1.35E+03	1.26E+03	1.43E-02	6.43E+02	**7.15E-03**
	*E*_*worst*_	1.59E+03	9.25E+02	1.49E+03	1.30E+03	1.99E-02	8.51E+02	9.95E-03
	*Std*.	3.58E+00	6.35E+00	6.69 E+01	1.01E+02	4.20E-03	3.69E+02	2.10E-03
*F*_8_	*E*_*best*_	2.04E+01	1.97E+01	1.94E+01	1.21E+01	1.05E+00	1.78E+01	1.92E+01
	*E*_*mean*_	2.57E+01	2.05E+01	2.04E+01	2.02E+01	**1.87E+01**	2.03E+02	2.01E+01
	*E*_*worst*_	2.72E+01	2.43E+01	2.23E+01	2.09E+01	2.06E+01	2.16E+02	2.03E+01
	*Std*.	1.87E+00	1.77E+01	1.54E+01	8.95E+00	5.56E+00	3.21E+00	9.88E-01
*F*_9_	*E*_*best*_	1.39E+01	1.98E+00	9.98E-01	6.93E-01	3.02E-03	4.16E-01	5.95E-03
	*E*_*mean*_	3.40E+02	4.73E+00	4.34E+00	3.06E+00	8.21E-03	1.84E+00	**6.94E-03**
	*E*_*worst*_	6.46E+02	7.95E+00	8.55E+00	6.96E+00	3.31E-02	4.17E+00	9.99E-03
	*Std*.	1.36E+01	1.44E+00	2.21E+00	1.70E+00	5.82E-03	1.01E+00	2.68E-03

The bold values provided in these tables denote the best value obtained by each method of the corresponding item.

**Table 6:** The statistical results regarding to *E*_*mean*_ gained by each algorithm for each benchmark.

Fun.	Items	Methods						
		SPSO 2011	QGA	EGPSO	FDE	GWO	IPSO	Proposed
*F*_10_	*E*_*best*_	1.59E+02	9.95E-01	3.94E+00	7.95E+00	5.49E-03	9.89E-03	1.67E-03
	*E*_*mean*_	4.73E+02	4.09E+00	8.88E+00	1.92E+01	7.59E-03	1.36E-02	**6.29E-03**
	*E*_*worst*_	8.05E+02	6.96E+00	1.79E+01	4.27E+01	2.35E-02	4.24E-02	9.98E-03
	*Std*.	1.67E+02	1.47E+00	2.93E+00	9.25E+00	4.29E-03	7.72E-03	2.56E-03
*F*_11_	*E*_*best*_	6.26E+00	5.99E+00	4.59E+00	4.32E+00	3.50E+00	1.98E+00	4.65E-01
	*E*_*mean*_	8.11E+00	7.62E+00	6.13E+00	5.71E+00	4.85E+00	4.63E+00	**4.27E+00**
	*E*_*worst*_	1.24E+01	9.17E+00	7.66E+00	7.57E+00	7.55E+00	5.98E+00	5.67E+00
	*Std*.	1.99E+00	7.32E-01	1.30E+00	1.44E+00	1.70E+00	5.78E-01	1.50E-01
*F*_12_	*E*_*best*_	8.97E+01	8.89E+01	6.66E+01	6.18E+01	9.36E-02	2.05E-02	5.52E-03
	*E*_*mean*_	1.09E+02	1.01E+02	8.59E+01	2.33E+01	2.18E+01	4.81E+00	**4.59E+00**
	*E*_*worst*_	2.95E+03	2.08E+03	7.86E+02	5.56E+02	2.33E+02	5.14E+00	3.76E+01
	*Std*.	1.85E+02	1.72E+02	1.61E+01	1.58E+01	6.22E+01	7.61E+01	9.25E+00
*F*_13_	*E*_*best*_	9.26E-01	4.58E-01	2.69E-01	3.65E-01	3.14E-03	3.10E-03	1.57E-03
	*E*_*mean*_	2.81E+00	1.02E+00	6.65E-01	6.49E-01	7.74E-03	7.45E-03	**3.87E-03**
	*E*_*worst*_	8.99E+00	1.36E+00	1.12E+00	9.65E-01	9.98E-03	9.94E-03	4.97E-03
	*Std*.	2.06E-01	2.11E-01	2.21E-01	1.52E-01	2.28E-03	2.19E-03	1.09E-03
*F*_14_	*E*_*best*_	3.03E+00	2.52E+00	2.16E+00	1.23E+00	3.65E-03	2.59E-03	2.55E-03
	*E*_*mean*_	3.92E+00	3.03E+00	2.98E+00	1.82E+00	1.16E-02	8.30E-03	**6.88E-03**
	*E*_*worst*_	4.48E+00	3.57E+00	3.55E+00	2.62E+00	3.38E-02	2.40E-02	1.03E-02
	*Std*.	3.96E-01	2.63E-01	3.79E-01	3.61E-01	7.44E-03	5.28E-03	2.43E-03
*F*_15_	*E*_*best*_	2.50E+02	2.69E+01	6.32E+01	8.98E+01	8.17E+01	4.17E+01	1.50E+01
	*E*_*mean*_	4.93E+02	3.15E+02	3.29E+02	3.53E+02	3.63E+02	3.21E+02	**3.04E+02**
	*E*_*worst*_	7.99E+02	4.31E+02	4.26E+02	4.29E+02	5.50E+02	4.25E+02	3.90E+02
	*Std*.	1.19E+02	1.43E+02	1.27E+02	1.01E+02	1.26E+02	1.37E+02	9.17E+01
*F*_16_	*E*_*best*_	5.88E+01	4.68E+01	3.54E+01	2.35E+01	6.44E+00	5.76E+00	4.65E+00
	*E*_*mean*_	3.23E+02	1.33E+02	1.15E+02	1.04E+02	7.72E+01	6.83E+01	**5.77E+01**
	*E*_*worst*_	7.48E+02	5.39E+02	4.98E+02	3.14E+02	2.12E+02	1.02E+02	9.88E+01
	*Std*.	1.97E+01	1.43E+01	1.26E+01	1.02E+01	1.44E+00	1.42E+00	1.34E+00
*F*_17_	*E*_*best*_	1.34E+02	1.02E+02	2.49E+01	1.78E+01	1.42E+01	1.35E+01	1.12E+01
	*E*_*mean*_	2.92E+02	1.55E+02	1.38E+02	1.20E+02	1.13E+02	1.08E+02	**1.03E+02**
	*E*_*worst*_	5.22E+02	5.14E+02	5.12E+02	4.23E+02	3.16E+02	2.52E+02	2.32E+02
	*Std*.	2.08E+01	2.78E+01	3.12E+01	1.98E+01	1.76E+01	1.72E+01	1.67E+01
*F*_18_	*E*_*best*_	2.45E+02	1.03E+02	1.12E+02	4.67E+01	4.35E+01	5.11E+01	1.32E+01
	*E*_*mean*_	1.03E+03	8.98E+02	9.62E+02	8.09E+02	7.28E+02	8.27E+02	**4.39E+02**
	*E*_*worst*_	3.78E+03	1.07E+02	1.15E+03	9.91E+02	9.89E+02	9.94E+02	5.07E+02
	*Std*.	4.46E+01	3.78E+01	4.33E+01	2.39E+01	2.01E+01	2.43E+01	1.13E+01
*F*_19_	*E*_*best*_	1.76E-09	1.55E-08	5.86E-10	1.92E-11	1.46E-11	6.56E-11	9.97E-12
	*E*_*mean*_	3.30E-09	2.74E-08	1.33E-10	7.46E-11	5.11E-11	8.76E-11	**5.07E-11**
	*E*_*worst*_	4.97E-09	3.86E-08	9.02E-10	9.95E-11	6.67E-11	9.94E-11	6.58E-11
	*Std*.	7.50E-09	6.47E-09	1.81E-10	2.44E-11	1.29E-11	9.93E-11	1.66E-11
*F*_20_	*E*_*best*_	8.47E+00	1.38E-01	9.63E-03	8.29E-03	1.27E-04	8.47E-04	9.10E-06
	*E*_*mean*_	3.20E+01	3.76E+00	3.74E+00	2.93E+00	1.48E-03	3.29E+00	**6.98E-04**
	*E*_*worst*_	5.67E+01	7.95E+00	6.96E+00	6.14E+00	1.47E-02	6.12E+00	6.96E-03
	*Std*.	1.48E+01	1.72E+00	2.01E+00	2.06E+00	3.44E-03	1.77E+00	1.85E-03

The bold values provided in these tables denote the best value obtained by each method of the corresponding item.

It is clear from Table 5, 6 that the proposed algorithm demonstrates superior performance over the majority of the 20 benchmark functions (specifically excluding *F*_4_ and *F*_8_) compared to the other six algorithms in terms of *E*_*mean*_. Note that the proposed algorithm is ranked the second among the seven algorithms when addressing *F*_4_ and *F*_8_. Thus, this overall analysis can be intuitively claimed that the proposed algorithm is highly promising over the most of the 20 test functions in terms of the solution optimality.

It is important to note that although ISAPSO performs well overall, one cannot strictly claim that it universally dominates its competitors across all 20 test functions. This is primarily due to the significant variability in the *E*_*mean*_ metric among different algorithms and benchmark functions. To rigorously quantify the significant improvements offered by the proposed ISAPSO, a statistical comparison needs to be conducted. In the conducted statistical comparison, a rank-based analysis is first executed to check the mean rank value of *E*_*mean*_ quality of each algorithm over the 20 test function. Based on the stochastic results displayed in Table 5, the rank value of *E*_*mean*_ obtained by each method for each test function and the average rank value regarding to *E*_*mean*_ of each method over the 20 test functions are summarized and displayed in Table 7.

**Table 7:** Rank values with respect to *E*_*mean*_ obtained by different methods for the 20 benchmarks.

	Methods						
Fun.	SPSO 2011	QGA	EGPSO	FDE	GWO	IPSO	Proposed
*F*_1_	7	5	3	6	4	2	1
*F*_2_	7	5	4	3	6	2	1
*F*_3_	5	7	4	6	2	3	1
*F*_4_	7	6	5	3	1	4	2
*F*_5_	7	6	5	4	3	2	1
*F*_6_	7	6	5	4	3	2	1
*F*_7_	7	4	6	5	2	3	1
*F*_8_	7	6	5	3	1	4	2
*F*_9_	7	6	5	4	2	3	1
*F*_10_	7	4	5	6	2	3	1
*F*_11_	7	6	5	4	3	2	1
*F*_12_	7	6	5	4	3	2	1
*F*_13_	7	6	5	4	3	2	1
*F*_14_	7	6	5	4	3	2	1
*F*_15_	7	2	4	5	6	3	1
*F*_16_	7	6	5	4	3	2	1
*F*_17_	7	6	5	4	3	2	1
*F*_18_	7	5	6	3	2	4	1
*F*_19_	6	7	5	3	2	4	1
*F*_20_	7	6	5	3	2	4	1
ave.	6.85	5.55	4.85	4.10	2.80	2.75	1.10

As shown in Table 7, the proposed ISAPSO achieves the highest average rank based on *E*_*mean*_ across the 20 benchmarks, followed by IPSO, GWO, FDE, EGPSO, QGA, and SPSO 2011. However, it is notable that this observation merely indicates the relative efficiency of ISAPSO,rather than concluding that ISAPSO performs significantly differently from or better than its counterparts. To address this, a non-parametric Friedman test (Demšar, 2006) is conducted at a 90% confidence level to evaluate the significance of the differences among the algorithms.Given that *K* = 7 algorithms are tested on *N* = 20 functions, the critical value *F*_*stastic*_ is calculated as 1.8265 using the MATLAB command *finv*(α, *K*−1, (*K*−1)(*N*−1)), where α = 0.1 represents the significance level. Based on the data in Table 7, the calculated Friedman statistic *F*_*s*_ is 80.8124, which is greater than the value of *F*_*stastic*_. Therefore, we can claim that the seven compared algorithms perform significantly different across the 20 benchmarks at the given confidence level in terms of *E*_*mean*_ metric (Demšar, 2006).

Although the non-parametric Friedman test confirms significant differences among the seven algorithms, it does not specify which algorithms differ from each other. To identify specific pairwise improvements, a *post-hoc* Bonferroni–Dunn test (Demšar, 2006) is conducted at a 90% confidence level. Given that *K* = 7 algorithms are evaluated on *N* = 20 test functions, the critical difference (*CD*) of pairwise *post hoc* Bonferroni–Dunn test equals to 1.6354 based on $q_{\alpha }\sqrt{\left(K\left(k+1\right)/\left(6N\right)\right)}$, where *q*_α_ is constantly set to be 2.394 (Demšar, 2006). Based on the summarized results shown in Table 7, the absolute differences in average ranks between ISAPSO and IPSO, GWO, FDE, EGPSO, QGA, and SPSO2011 are 1.65, 1.70, 3.00, 3.75, 4.45, and 5.75, respectively. Since all these values are greater than that of the critical difference of pairwise *post hoc* Bonferroni–Dunn test (i.e., *CD* = 1.6354), it can be sufficiently conclusive that the proposed ISAPSO significantly dominates its six contenders over the 20 test functions at the confidence level of 90% in terms of the average solution optimality. Thus, the proposed ISAPSO algorithm can be efficient in solving different global optimization issues.

### 5.3 Verification of the ISAPSO-based path planner

After verifying the proposed ISAPSO via the benchmark study conducted in the above subsection, this subsection evaluates the performance of the ISAPSO-based path planning method on the UAV 3D path planning issue. For a rigorous comparison, the performance of the ISAPSO-based path planning approach is compared with those of the same six evolutionary algorithms depicted in SubSection 5.2. In each simulation scenario, the swarm size and maximum iteration number of each approach are empirically set to be 40 and 100, respectively. The rest simulation parameters for each approach are referred to SubSection 5.2. For a comprehensive comparison and reducing impacts of randomness, a Monte-Carlo experiment with 30 runs is conducted in each simulation scenario for each considered method. Moreover, the fitness value (*FV*) and the computation time (*CT*) of each method are reported in each numerical simulation in order to quantitatively assess the performance of each approach.

In each numerical simulation, the number of waypoints is set to be 5 to compromise the solution optimality and computation time. The physical constraints of the UAV in the two numerical simulations are displayed in Table 8. The first numerical simulation is conducted in a 100km × 100km × 100km workspace with 10 mountains in the case where the UAV is navigated from the start point (1, 1, 1) to the destination point (100, 100, 40). The second scenario simulates to navigate the UAV from the start point (10, 10, 0) to the destination point (200, 200, 100) in a 200km × 200km × 200km workspace owing 12 mountains. The terrain information of each mountain in the two conducted simulations is illustrated in Tables 9, 10.

**Table 8:** The physical constraints of the UAV in the two numerical simulations.

Numerical simulation	Physical constraints		
	*L*^*max*^ (km)	ψ^*max*^ (°)	θ^*max*^ (°)
1	200	65	65
2	380	75	75

**Table 9:** The terrain information of each mountain in the first numerical simulation (where mn denotes mountain number).

mn	(x_*i*_,*y*_*i*_) (km)	(x_*si*_,*y*_*si*_) (km)	z_*i*_ (km)
1	(78.51, 48.80)	(5.70, 5.70)	61.79
2	(82.04, 78.74)	(7.85, 7.85)	60.12
3	(95.61, 82.73)	(3.76, 3.76)	79.39
4	(51.19, 67.27)	(3.35, 3.35)	62.15
5	(38.29, 86.73)	(9.04, 9.04)	31.10
6	(26.24, 73.52)	(4.52, 4.52)	65.01
7	(65.72, 29.77)	(7.19, 7.19)	76.98
8	(24.47, 24.51)	(3.13, 3.13)	40.67
9	(54.81, 86.57)	(6.64, 6.64)	73.21
10	(89.11, 27.81)	(3.75, 3.75)	93.56

**Table 10:** The terrain information of each mountain in the second numerical simulation (where mn denotes mountain number).

mn	(x_*i*_,*y*_*i*_) (km)	(x_*si*_,*y*_*si*_) (km)	z_*i*_ (km)
1	(168.87, 132.27)	(9.35, 9.35)	85.60
2	(181.84, 44.58)	(8.35, 8.35)	128.58
3	(196.58, 154.03)	(12.59, 12.59)	130.06
4	(49.53, 149.11)	(7.01, 7.01)	65.94
5	(123.46, 55.47)	(17.44, 17.44)	174.54
6	(155.59, 63.97)	(13.26, 13.26)	152.34
7	(195.67, 143.83)	(12.35, 12.35)	172.04
8	(109.18, 172.05)	(7.86, 7.86)	71.68
9	(67.74, 102.55)	(17.24, 17.24)	176.39
10	(49.67, 103.88)	(11.83, 11.83)	133.76
11	(145.09, 140.47)	(12.04, 12.04)	100.87
12	(42.47, 197.45)	(7.48, 7.48)	83.40

**Figure 9:**
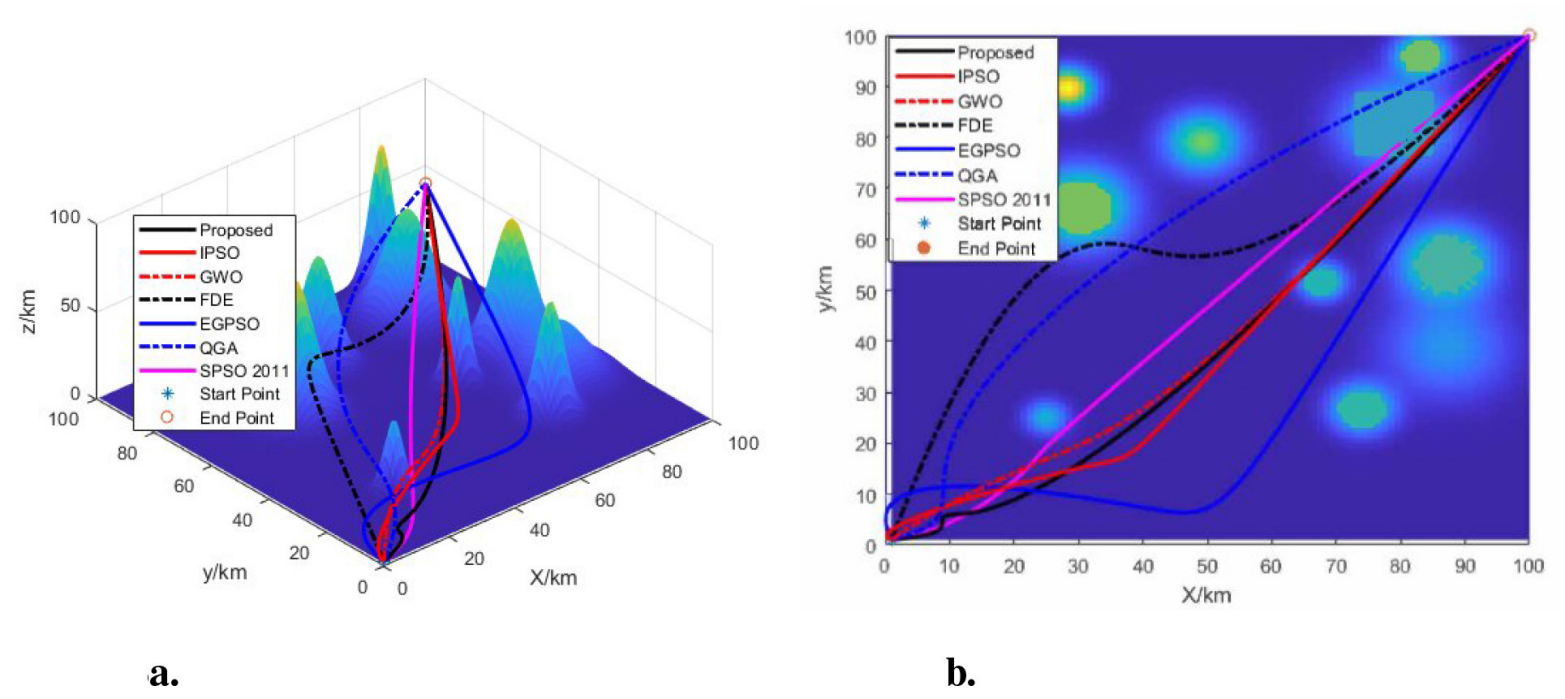
The best generated path of each method in the first numerical simulation. **(a)** 3D path. **(b) 2D** overlook path.

**Figure 10:**
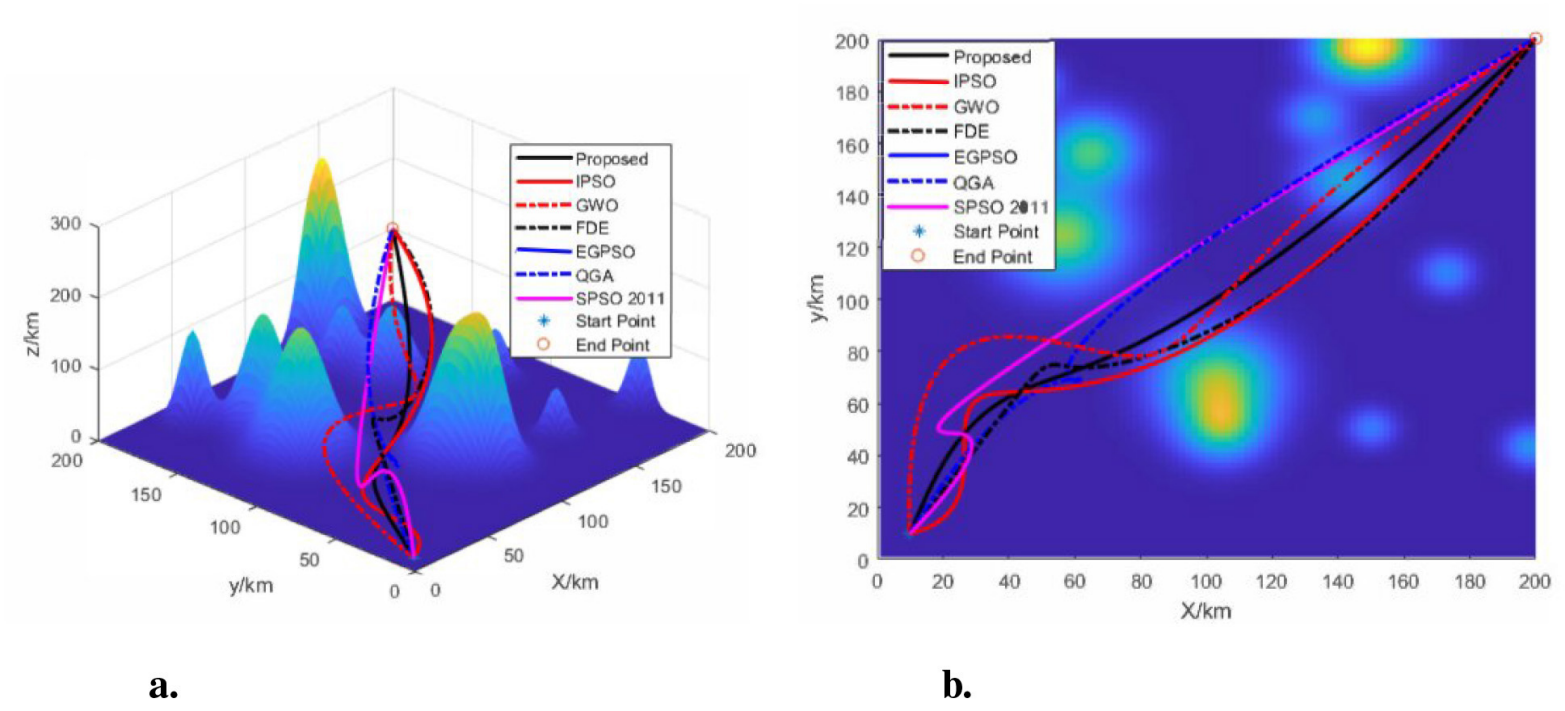
The best generated path of each method in the second numerical simulation. **(a)** 3D path. **(b)** 2D overlook path.

**Figure 11:**
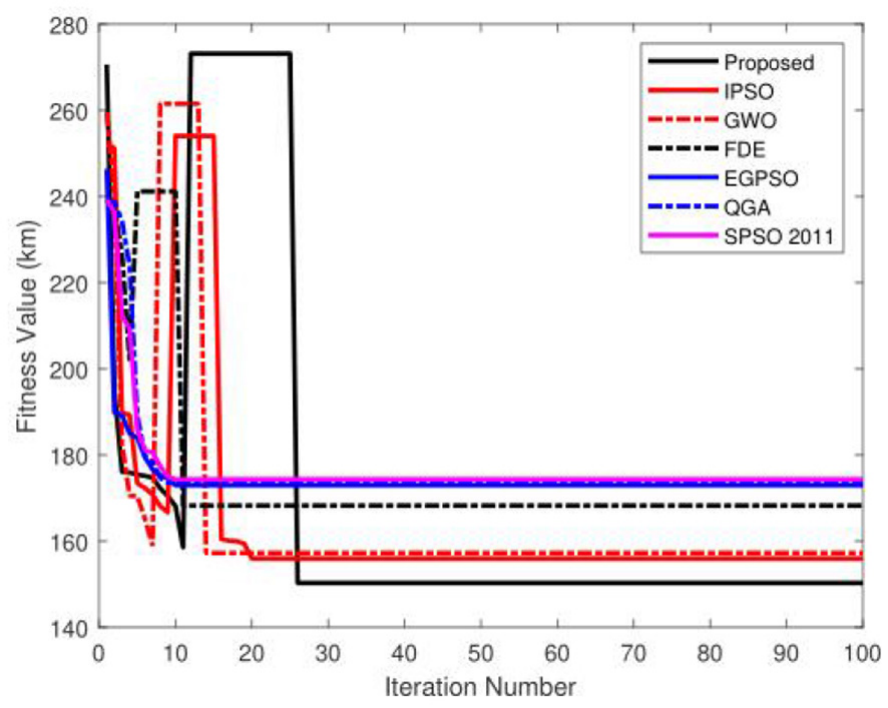
The fitness curve corresponding to the best generated path for each method in the first simulation.

**Figure 12:**
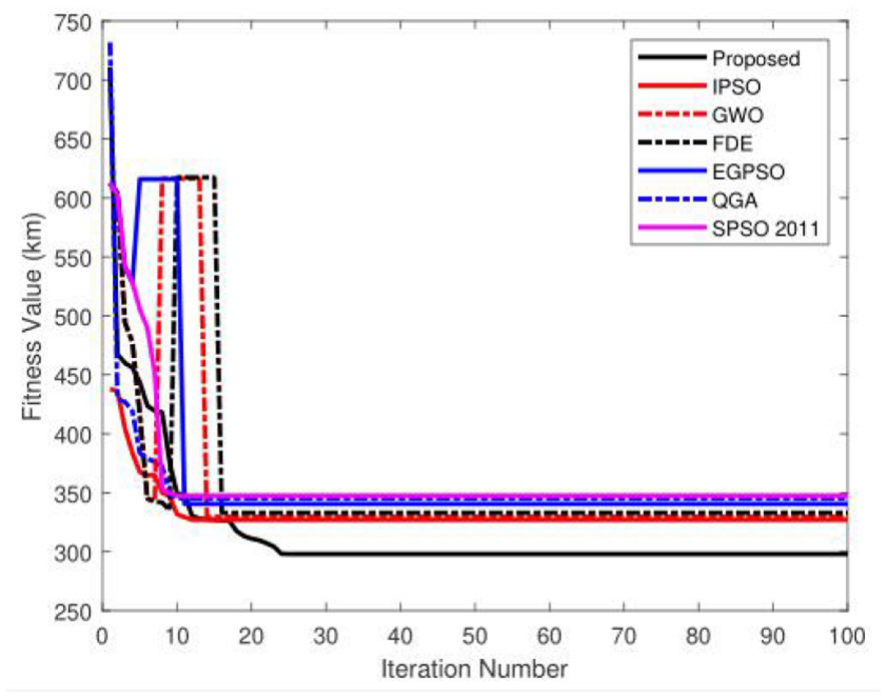
The fitness curve corresponding to the best generated path for each method in the second simulation.

After conducting the described Monte-Carlo experiment for each simulation scenario, the statistic results regarding *FV* and *CT* gained by each approach are summarized in Tables 11, 12. The best generated path yielded by each method among the Monte-Carlo study for the two numerical simulations is demonstrated in Figures 9,10. The fitness curve corresponding to the best generated path for each studied case is illustrated in Figures 11, 12.

**Table 11:** The statistical results of *FV* and *CT* gained by approach in the first simulation.

Item	Results	Methods						
		SPSO 2011	QGA	EGPSO	FDE	GWO	IPSO	Proposed
FV (km)	*Best*	174.35	173.29	173.01	168.24	157.24	155.95	150.30
	*Mean*	185.63	182.35	178.49	175.72	162.43	159.88	**154.37**
	*worst*	193.35	191.77	190.46	189.55	170.13	168.47	160.38
	*Std*.	9.02	8.78	8.65	7.85	7.27	6.14	5.03
*CT*(s)	*Best*	13.11	19.67	17.92	18.55	16.77	16.44	14.44
	*Mean*	**14.52**	20.88	18.37	19.36	17.86	17.22	15.36
	*worst*	15.33	21.38	19.53	20.16	18.92	18.42	16.27
	*Std*.	0.87	0.92	0.96	0.94	0.97	0.89	0.88

The bold values provided in these tables denote the best value obtained by each method of the corresponding item.

**Table 12:** The statistical results of *FV* and *CT* gained by approach in the second simulation.

Item	Results	Methods						
		SPSO 2011	QGA	EGPSO	FDE	GWO	IPSO	Proposed
*FV*(km)	*Best*	347.46	345.26	340.51	332.92	329.57	327.14	298.07
	*Mean*	353.35	351.35	346.46	339.05	333.96	332.09	**330.12**
	*worst*	364.45	363.24	352.49	345.72	344.90	341.05	337.55
	*Std*.	6.23	6.03	6.11	5.94	6.47	5.98	4.36
*CT*(s)	*Best*	18.87	25.78	22.35	23.07	20.41	20.23	19.02
	*Mean*	**19.48**	26.57	23.33	24.12	21.38	21.07	20.79
	*worst*	20.36	27.08	24.02	25.86	22.39	22.47	21.38
	*Std*.	0.89	0.91	0.86	0.90	0.84	0.85	0.90

The bold values provided in these tables denote the best value obtained by each method of the corresponding item.

It is apparent from Figures 9, 10 that each considered approach can generate a collision-avoidance flyable path for the UAV in each numerical simulation. This can reflect the feasibility and efficiency of each method over the UAV 3D path planning issue to some extent. However, it is obvious from Tables Tables 11-12 that the developed ISAPSO-based path planning approach dominates the six compared approaches in terms of the average fitness value in the two numerical simulations, which can confirm that the developed approach performs generally superior to its six peers in handling UAV 3D path planning problem. Moreover, it can be also observed from Tables 11, 12 that the developed path planner can gain the the best values regarding to the best and worst fitness values among the seven considered approaches in the Monte-Carlo experiment conducted for the two numerical simulations. This could further reflect the high efficiency and performance of the developed path planning approach.

Moreover, it can be easily inferred from Tables 11, 12 that SPSO 2011 and the ISAPSO-based planning method are respectively ranked the first and second among the seven approaches in the Monte-Carlo experiment conducted for the two numerical simulations in terms of the computation time.This indicates that the computation complexity of SPSO 2011 and ISAPSO-based approach are cheaper than those of the other compared approaches. It is important to note that although SPSO 2011 is computationally cheaper than our developed path planning approach probably due to the fact that a novel self-adaptive parameter updating strategy is adopted in ISAPSO, the distinction of the computation time between SPSO 2011 and ISAPSO-based method is not outstanding, which could be neglected since the path planning issue considered in this paper is a global path planning issue, which requires to generate path off-line.

It is notable from Figures 11, 12 that the fitness curves of some approaches keep raising in the early of the evolution process and maintain dropping or stable in the latter evolution stage. This changing tendency of the fitness curve could be likely interpreted by the fact that the self-adaptive constraint handling technology is implemented in these path planning methods. Since both unfeasible and feasible solutions are allowed to participate into the next iterations in the early evolution stage via our developed constraint handling technology, the feasible solution with greater fitness value would dominate the unfeasible solution having smaller fitness value, which thus leads the fitness curves of some approaches to raising in the early evolution. In the latter evolution stage, since the redefinition of feasible solution becomes stricter though the developed constraint handling technology, only the real feasible solutions are permitted to join in the next iteration, which results in the feasible solution having smaller fitness value vanquishing the one with bigger fitness value. Probably due to this mechanism, as displayed in Figures 11, 12, the fitness curves of some considered approaches keep dropping or stable in the latter of evolution process.

To summarize the analysis described above, it can be conclusive that the developed ISAPSO-based path panning approach is highly powerful and promising in solving UAV 3D path planning problem in terms of the solution optimality. Moreover, the computation complexity of this developed approach is highly competitive and comparable with those of its peer. Thus, the developed ISAPSO-based path planning method could be regarded as a paramount alternative in the field of UAV path planning.

## 6 Conclusion and future work

To efficiently handle the UAV 3D path planning problem, this paper proposes a novel ISAPSO algorithm based on SPSO 2011 and EGT. For well balancing the global and local search abilities of particles, a novel self-adaptive parameter updating principle inspired by the ESS of EGT hyperbolic tangent function is proposed in ISAPSO to tune the three key control parameters of the particle. Moreover, leveraging the proposed PSO algorithm, a new ISAPSO-based path planing approach is completed in this paper. Aiming at easily handling constraints of the path planning problem and diversifying the particle swarm, a modified self-adaptive constraint handling technology according to the feasibility-based principle is adopted in the ISAPSO-based planner. Finally, the performance of the proposed ISAPSO algorithm is assessed through a benchmark study over 20 benchmark test functions. Followed the benchmark study, the ISAPSO-based path planning method is evaluated under different simulation scenarios. The simulation results regarding to the benchmark study reveal that the proposed ISAPSO algorithm performs significantly better than its peers at the confidence level of 90% over the 20 selected test functions. Moreover, the ISAPSO-based path planning method generally dominates its competitors in terms of the path optimality.

The results and method shown in this paper raise some works deserved future study. Firstly, as a stochastic algorithm, the convergence issue of the proposed PSO remains unaddressed. To fully guarantee convergence, an analytical investigation of the algorithm is needed in the near future. Secondly, the path planning issue considered in this paper belongs to the community of global path planing. The feasibility and efficiency of the developed path planning approach over online path planning problem with uncertain or moving obstacles could be considered in the near future. Next, this paper mainly devotes its interest on single UAV path planning. As an extended study of this paper, we are considering the possibility of applying the developed approach to handle multiple-UAVs cooperative path planning problem. Last but not least, since the proposed method is a heuristic algorithm with swarm-based nature, the proposed method could be considered to solve the multiple-objective path planning issue by adopting the non-dominated definition of Pareto front.

## Data Availability

The original contributions presented in the study are included in the article/Supplementary material, further inquiries can be directed to the corresponding author.
