# Supplementary material for: A bionic intelligent method combining evolutionary game theory with particle swarm optimization for UAV 3D path planning
Source: Front Neurorobot. 2026 Jun 29;20:1857152. doi: 10.3389/fnbot.2026.1857152 (PMC13357615; doi:10.3389/fnbot.2026.1857152)
Supplement: Supplementary file 1 [file Supplementary_file_1.pdf]

A bionic intelligent method combining evolutionary game theory with particle swarm optimization for UAV 3D path planning

Lixin Jia [1] **and Peng Shi** [1,*]

[1] *School of Astronautics, Beihang University, 37 Xueyuan Road, Haidian District, Beijing, 100191, PR China*

Correspondence*:
Corresponding Author:Peng Shi
shipeng@buaa.edu.cn

APPENDIX

This appendix shows the average fitness curves of different algorithms over different test functions shown in Subsection 5.2.

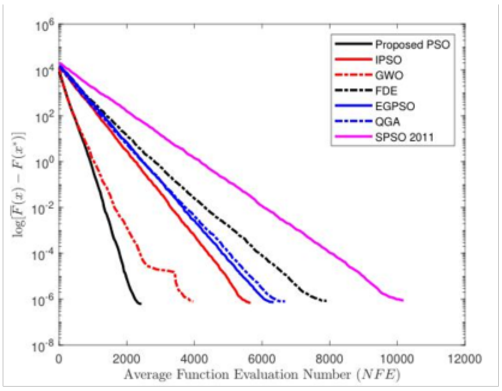

(a)F1

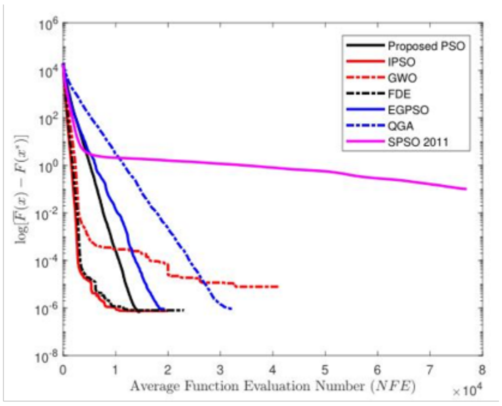

(b)F2

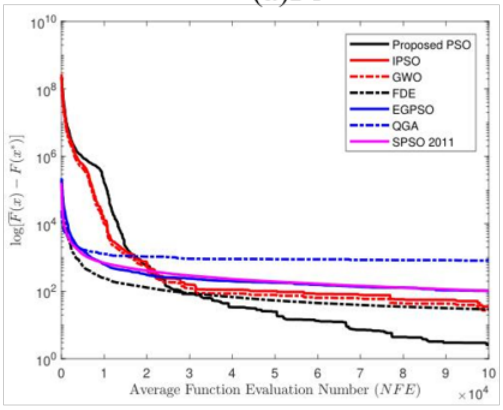

(c)F3

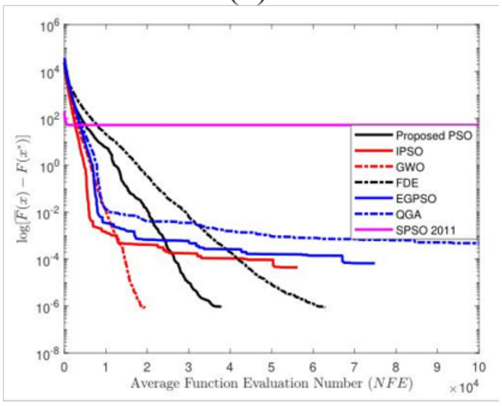

(d)F4

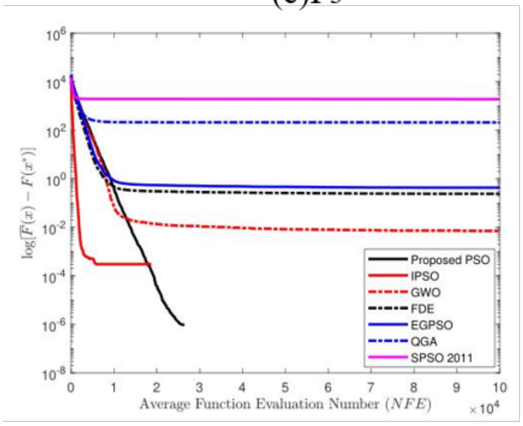

(e)F5

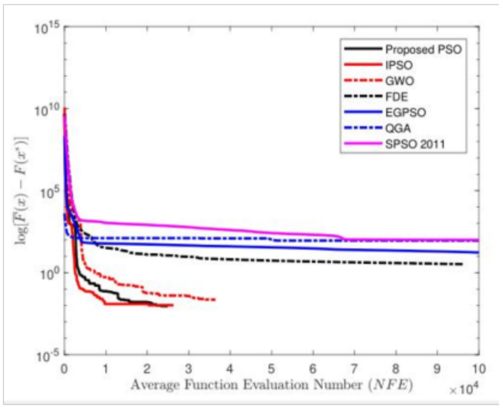

(f)F6

Supplmentary Figure 1. Convergence curves of E_{mean} obtained by different algorithms over test functions F_1-F_6

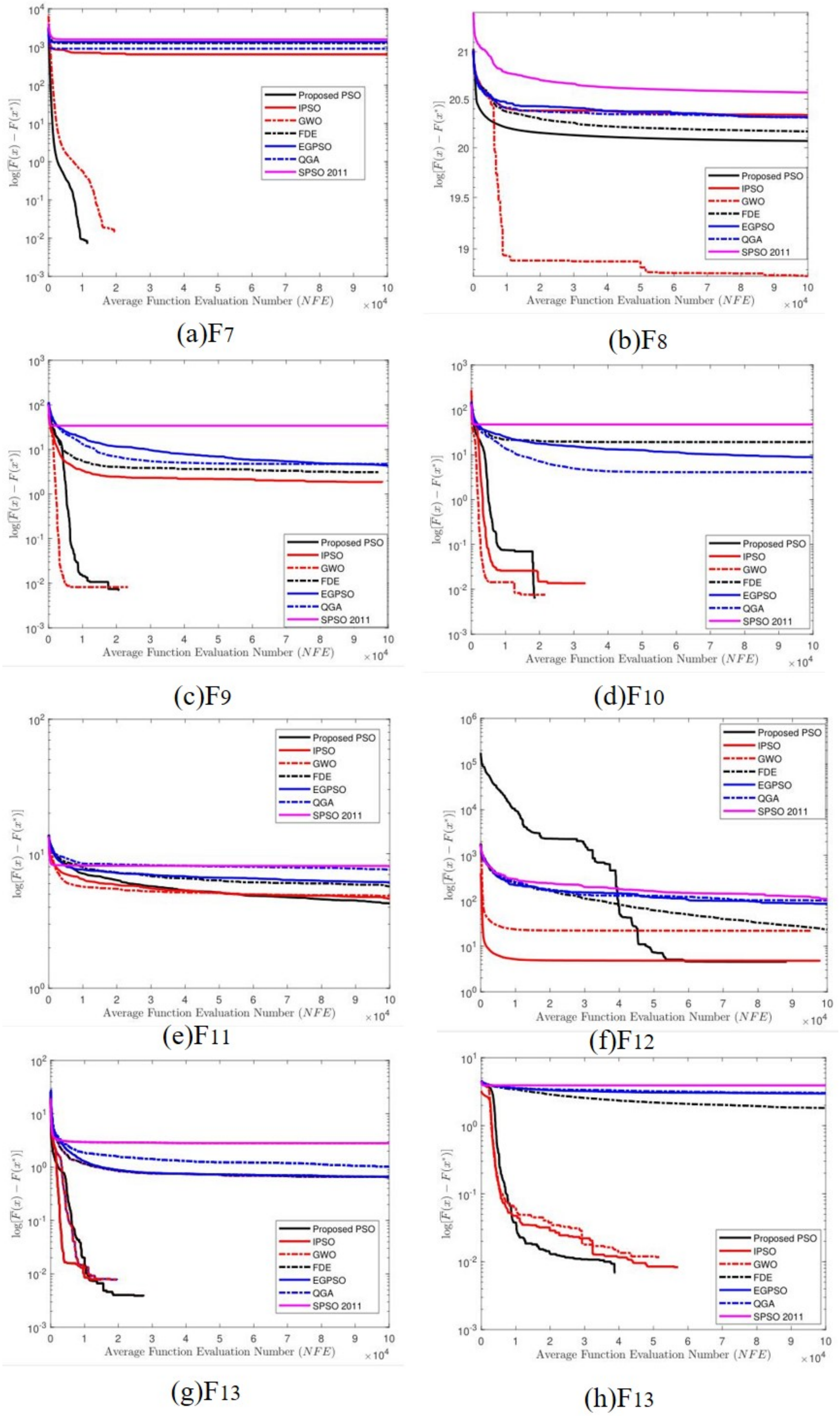

Supplementary Figure 2. Convergence curves of E_{mean} obtained by different algorithms over test functions F_7-F_{14}

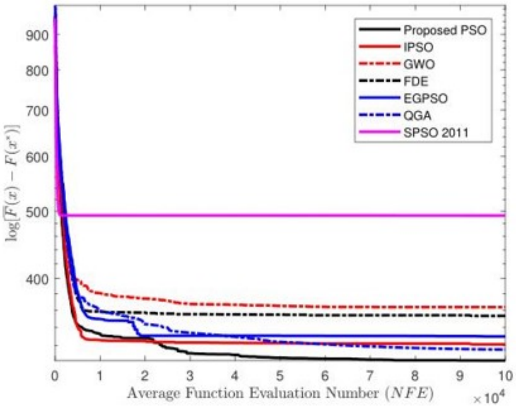

(a)F15

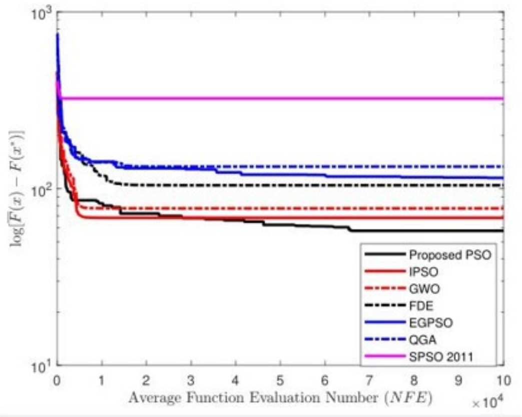

(b)F16

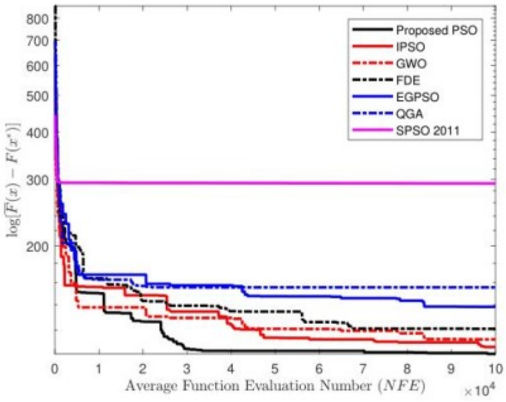

(c)F17

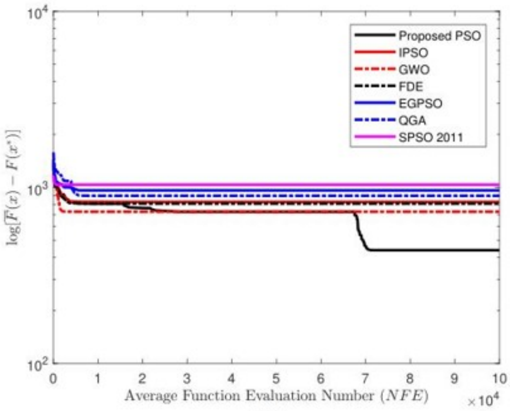

(d)F18

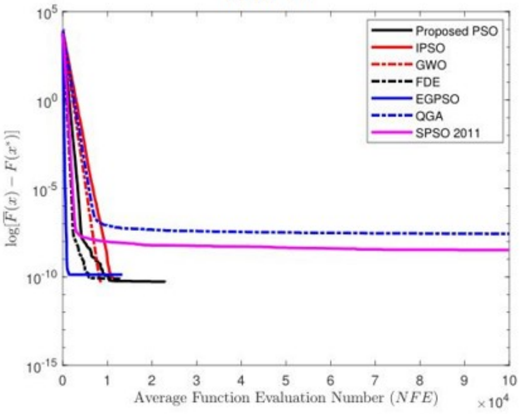

(e)F19

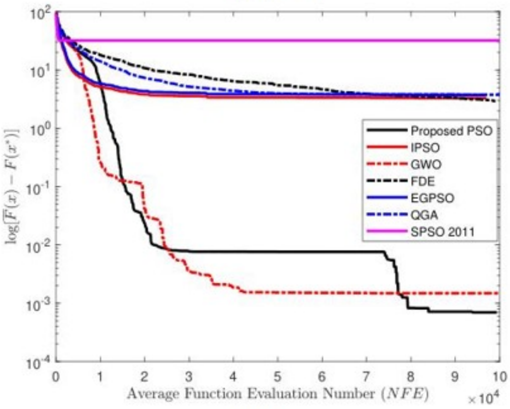

(f)F20

Supplementary Figure 3. Convergence curves of E_{mean} obtained by different algorithms over test functions F_{15}-F_{20}
